# Sirtuin Modulators in Cellular and Animal Models of Human Diseases

**DOI:** 10.3389/fphar.2021.735044

**Published:** 2021-09-28

**Authors:** Jun Young Hong, Hening Lin

**Affiliations:** ^1^ Department of Chemistry and Chemical Biology, Cornell University, Ithaca, NY, United States; ^2^ Department of Chemistry and Chemical Biology, Howard Hughes Medical Institute, Cornell University, Ithaca, NY, United States

**Keywords:** sirtuin, inhibitor, activator, cancer, neurodeganaration, cardiovacsular diseases, SIRT1, SIRT2

## Abstract

Sirtuins use NAD^+^ to remove various acyl groups from protein lysine residues. Through working on different substrate proteins, they display many biological functions, including regulation of cell proliferation, genome stability, metabolism, and cell migration. There are seven sirtuins in humans, SIRT1-7, each with unique enzymatic activities, regulatory mechanisms, subcellular localizations, and substrate scopes. They have been indicated in many human diseases, including cancer, neurodegeneration, microbial infection, metabolic and autoimmune diseases. Consequently, interests in development of sirtuin modulators have increased in the past decade. In this brief review, we specifically summarize genetic and pharmacological modulations of sirtuins in cancer, neurological, and cardiovascular diseases. We further anticipate this review will be helpful for scrutinizing the significance of sirtuins in the studied diseases.

## Introduction

Sirtuins, the class III histone deacetylase, use NAD^+^ to remove various acyl modifications on protein lysine residues (Sauve et al., 2001; Sauve et al., 2006; Feldman et al., 2012). In humans, there are seven sirtuins (SIRT1-7), with different acyl group specificities and subcellular localizations. Through deacylation, sirtuins regulate a wide range of biological functions, such as cell proliferation, metabolism, transcription, apoptosis, and cell signaling (Avalos et al., 2005; Machado de Oliveira et al., 2012; Morris, 2013; Hu et al., 2014; Jing and Lin, 2015; Bheda et al., 2016; Blank et al., 2017; Cha et al., 2017; Kosciuk et al., 2019; Wang and Lin, 2021). Consequently, sirtuins have been linked to various diseases, including cancer, neurological, and cardiovascular diseases (Borradaile and Pickering, 2009; Haigis and Sinclair, 2010; Hu et al., 2014; Fujita and Yamashita, 2018). Many sirtuin activators and inhibitors have been designed and used in cellular and animal studies. In this review, we summarized sirtuin modulators that showed promising therapeutic effects in cancer, neurological, and cardiovascular disease models.

The seven mammalian sirtuins localize to different cellular compartments. SIRT1, SIRT6, and SIRT7 mainly reside in the nucleus, but SIRT1 and SIRT6 are also found in the cytoplasm. SIRT2 is mainly in the cytoplasm. SIRT3, SIRT4, and SIRT5 are primarily located in the mitochondria (Feldman et al., 2012). Yet, under certain conditions, like cell division or stress, several sirtuins may change their cellular locations (North and Verdin, 2007; Tanno et al., 2007; Nishida et al., 2015).

**FIGURE 1:**
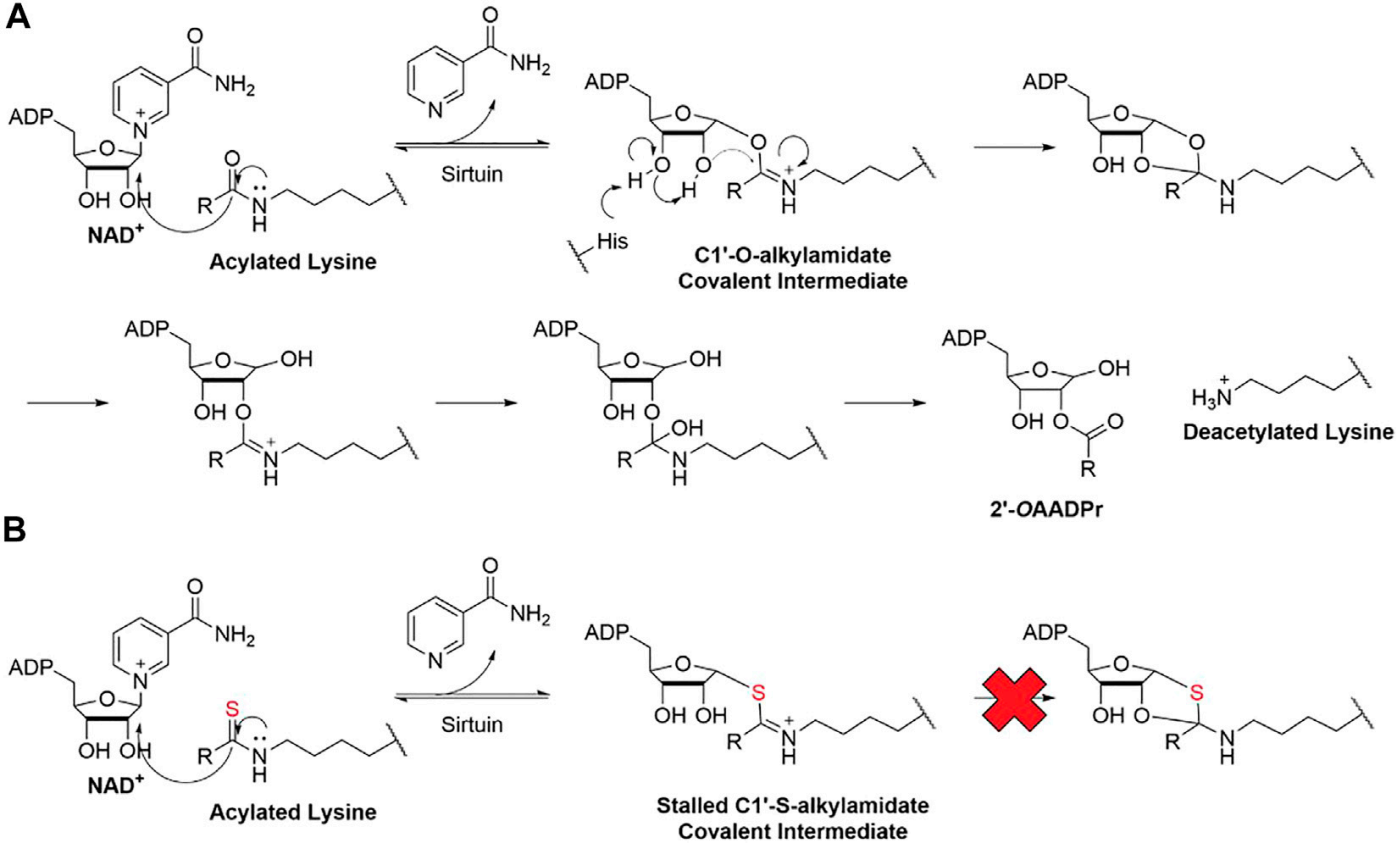
**(A)** Schematic summary of sirtuin deacylation mechanism. **(B)** Schematic summary of mechanism-based inhibition of sirtuin

All seven sirtuins use a similar mechanism to catalyze lysine deacylation (Avalos et al., 2005; Feldman et al., 2015; Wang and Lin, 2021). First, the amide group of the acyl lysine attacks C1 of the NAD^+^ ribose and releases nicotinamide (Figure 1). This forms a covalent C1′-O-alkylamidate intermediate. Then, the conserved histidine deprotonates the 3′-hydroxyl group of the NAD^+^ ribose, which deprotonates the 2′-hydroxyl group. The deprotonated 2′-hydroxyl group attacks the C1′-O-alkylamidate intermediate, forming a 1′,2′-cyclic intermediate. The acyl group is transferred to the 2′-hydroxyl group and releases the deacylated lysine product and 2′-O-acyl ADP-ribose (Avalos et al., 2005; Feldman et al., 2015; Wang and Lin, 2021).

Even though SIRT1-7 operate through a similar catalytic mechanism, different sirtuins prefer different acyl substrates due to differences in their substrate pockets. SIRT1 and SIRT3 remove acetyl and long-chain fatty acyl groups from lysine *in vitro*, but so far known physiological substrates are all deacetylation substrates (Feldman et al., 2012; Teng et al., 2015). SIRT2 removes acetyl, long-chain fatty acyl, 4-oxononanoyl, and benzoyl groups (Feldman et al., 2012; Teng et al., 2015; Jin et al., 2016; Huang et al., 2018). SIRT4 removes lipoyl, biotinyl, methylglutaryl, hydroxymethylglutaryl, and 3-methylglutaconyl groups (Mathias et al., 2014; Anderson et al., 2017; Kumar and Lombard, 2017; Pannek et al., 2017). SIRT5 removes charged malonyl, succinyl, and glutaryl groups (Du et al., 2011). SIRT6 and SIRT7 remove acetyl and long-chain fatty acyl groups (Zhang et al., 2016; Tong et al., 2017). Both acetyl and long-chain fatty acyl substrates were known for SIRT6, but only physiological acetyl substrates are known for SIRT7.

Many modulators are strategically designed to target different sirtuins and generate beneficial effects in human disease models. As many recently published research articles emphasized the importance of sirtuins in cancer, neurological, and cardiovascular diseases, we have specifically chosen these in this review. We summarize the various sirtuin modulators that have been developed, focusing on those that have demonstrated biological effects in cellular or animal models. Because one common concern about small molecules is whether their biological activity is through on-target effect or not, we will emphasize whether the sirtuin modulators’ biological activity is confirmed by other means, such as knockdown, knockout, or overexpression of the sirtuin being targeted. Accordingly, we will spend more attention describing the sirtuin modulators for which the biological activity has been confirmed by other methods.

Previous reports focused on analyzing the roles of sirtuins and a few selected modulators (Chalkiadaki and Guarente, 2015; Gomes et al., 2019). Thus, our review with a more extensive summary of direct sirtuin modulators can help the readers to choose a suitable compound for their experiments. In addition to Table 1 with the compound structure, inhibition profile, and results from biological studies, we added tables that list cancer cell lines affected by the sirtuin modulators (Tables 2–6). This way, this review can serve as an initial useful guideline for those thinking of using sirtuin modulators in their studies.

**TABLE 1:** Summary of the sirtuin modulators and its biological assessment in cellular and animal studies.

Compound	Structure	Modulation profile	Cellular studies	Animal studies	References
EX-527/Selistat	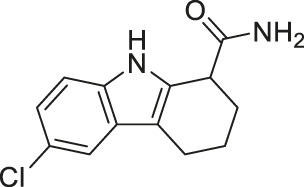	Inhibits SIRT1 with 200-fold selectivity over SIRT2	- General: increases several SIRT1 deacetylation targets, including p53, NBS, and Rad1	- HHUA endometrial carcinoma tumor xenograft mice: decreased tumor growth	(Napper et al., 2005; Solomon et al., 2006; Gertz et al., 2013; Asaka et al., 2015; Kim et al., 2015; Oon et al., 2015; Kim et al., 2016; Chen et al., 2017; Chen et al., 2019; Li et al., 2019; Muscolini et al., 2019; Nikseresht et al., 2019; Yousafzai et al., 2019; Wang et al., 2020; Wei et al., 2021)
			- U87MG and LN0299 glioma: decreased proliferation and colony formation through increasing p53 and ac-p53 levels, and caspase activation	- A549 lung tumor xenograft mice: decreased tumor growth with MK-1775 combination	
			- 5637 and T24 bladder: decreased proliferation, glycolysis, and glucose uptake (Opposite results from SIRT1 overexpression)	- PANC-1 pancreatic tumor xenograft mice: increased tumor growth and showed no synergistic effect with gemicitabine	
			- H460-R cisplatin-resistant lung cancer: increased sensitivity to cisplatin (Opposite results from SIRT1 overexpression; Confirmed by SIRT1 knockdown)	- Single prolonged stress mice mimicking post-traumatic stress disorder: hindered expression of MAO-A, stabilized serotonin, and ensured normal neuronal plasticity (*Sirt1* deleted mice had less anxiety and freezing time)	
			- HEC151, HEC1B, and HHUA endometrial carcinoma: decreased proliferation (Opposite results from SIRT1 overexpression)	- Morphine addicted mice: increased SIRT1 expression and alleviated morphine addiction	
			- PC-3 prostate: increased the effect of vesicular stomatitis virus oncolysis (Confirmed by SIRT1 knockdown)	- Rat model of middle cerebral artery occlusion: improved the survival rate, and decreased infarction volume.	
			- Chemo-resistant stem-like cells from leukemia K562 cells: increased the anticancer effect of 17-AAG and AUY922 (Confirmed by SIRT1 knockdown)	—	
			- A549 lung: showed synergistic antiproliferative effect with MK-1775 (through increasing ac-Rad51 and NBS1)	—	
			- PANC-1 pancreatic: decreased proliferation and increased the anticancer effect of gemcitabine (Confirmed by SIRT1 knockdown)	—	
			- P19 embryonic carcinoma cell: promoted differentiation into neuronal cells (Confirmed by SIRT1 knockdown)	—	
AGK2	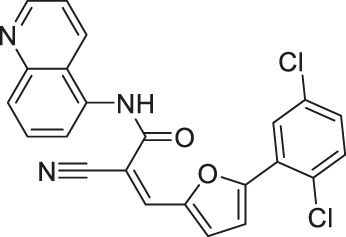	Inhibits SIRT2 with 5-fold selectivity over SIRT1	- GB2, GB3, GB11, and GB16 glioblastoma: decreased cell proliferation (did not show antiproliferative effect in SIRT2 knockdown GB2 cells) (SIRT2 knockdown GB2 and GB16 cells proliferated slower)	- Drosophila model of Parkinson’s Disease: rescued the decrease of dorsomedial neurons	(Outeiro et al., 2007; Wang et al., 2016; Funato et al., 2018; She et al., 2018; Kaitsuka et al., 2020; Yang et al., 2020)
			- HCT-116 colorectal: decreased effects of cisplatin, 5-FU, oxaliplatin, gefitinib, LY294002, and metformin	- Lipopolysaccharides-induced brain injury mice: lowered neuroinflammation and TUNEL signal	
			- SW620 colorectal: increased effects of cisplatin, 5-FU, oxaliplatin, gefitinib, LY294002, and metformin	Middle cerebral artery occlusion mice: decreased apoptosis	
			- Human neuroglioma cells (H4): decreased α-Synuclein-mediated toxicity (Consistent with SIRT2 knockdown)	—	
			- Cultured hippocampal neurons: protected cell deaths from H_2_O_2_ and stimulated neuroprotection (Consistent with SIRT2 knockout DT40 cells)	—	
AK7	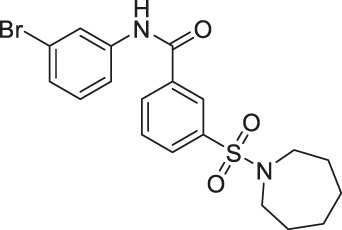	Inhibits SIRT2	—	- GB2 tumor xenograft mice: decreased tumor growth (Mice with SIRT2 knockdown GB2 cells survived longer and had less tumorigenicity)	(Taylor et al., 2011; Funato et al., 2018; Wu et al., 2018; Wu et al., 2020)
				- Middle cerebral artery occlusion mice: decreased infarction volume and promoted neurological recovery through pP38 activation (SIRT2 knockdown neuro-2a cells activated pP38)	
				- Sevoflurane-treated neonatal rat: decreased pro-inflammatory marker and increased anti-inflammatory marker	
SirReal2	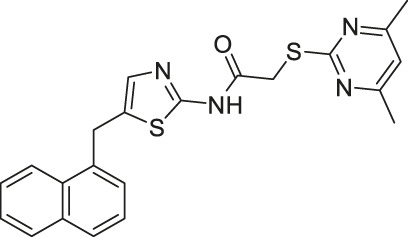	Inhibits SIRT2	- HGC-27 and MGC-803 gastric: decreased proliferation and migration (SIRT2 knockdown had less migration) (Mice with SIRT2 knockdown showed less metastatic tumors and tumor growth)	—	(Rumpf et al., 2015; Li et al., 2018; Spiegelman et al., 2018)
			- MCF7, MDA-MB-231, MDA-MB-468 breast, HCT-116, HT-29, SW948 colorectal, A549, H520 lung, K562 lymphoma, HeLa cervical: decreased cell proliferation		
RK-91230156	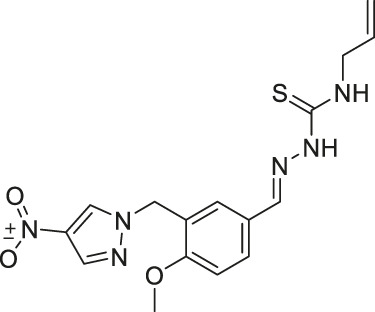	Inhibits SIRT2	- MCF7 breast: decreased cell proliferation through degradation of c-Myc and increased acetylated eIF5a	—	Shah et al. (2016)
NCO-90/141	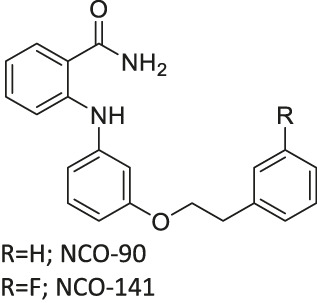	Inhibits SIRT2	- HTLV-1-trasnformed T-cells: induced autophagic cell death and increased mitochondrial superoxide level	- Senesce-accelerated mouse prone-8 mice: increased spatial learning and memory deficiency of 5 month-old mice; did not have any effects on 8 month-old mice (proved SIRT2 inhibition in hippocampus by monitoring the elevated level of Abca1	(Suzuki et al., 2012; Kozako et al., 2018; Diaz-Perdigon et al., 2020)
			- S1T, MT-2, Jurkat, and HL60 leukemia cells: increased acetylation of histone H4, but did not alter acetylation of p53		
KPM-2	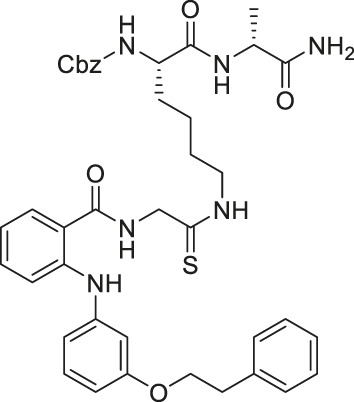	Inhibits SIRT1, SIRT2, and SIRT3	- MDA-MB-231 breast: decreased proliferation (usage of SIRT1 selective inhibitor with a similar structure as KPM-1 showed no effect; usage of less potent SIRT2 inhibitor with a similar structure showed weaker cytotoxicity)	—	Mellini et al. (2017)
			- Neuro-2a: promoted neurite outgrowth		
Compound 53	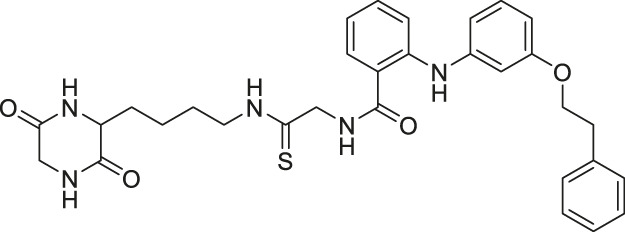	Inhibits SIRT2	- Neuro-2a: promoted neurite outgrowth	—	Mellini et al. (2019)
			- MCF7 breast: decreased proliferation and increased acetyl α-tubuliin		
NPD11033	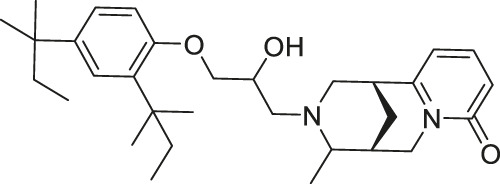	Inhibits SIRT2	- PANC-1 pancreatic: decreased proliferation and increased acetylated eIF5a (SIRT2 knockdown decreased proliferation) (inactive analog RK-0310020 did not have any affect)	—	Kudo et al. (2018)
Compound 6f	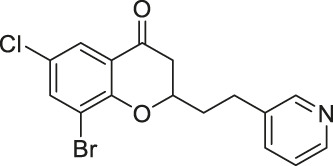	Inhibits SIRT2	- MCF7 breast, and A549 lung: decreased proliferation and arrested G1/G0 phase cell cycle arrest (increased acetyl α-tubulin in MCF7)	—	Seifert et al. (2014)
Compound 12a	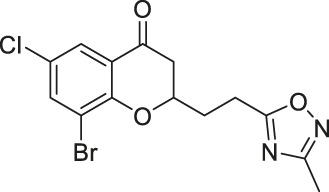	Inhibits SIRT2	MCF7 breast, and A549 lung: decreased proliferation and arrested G1/G0 phase cell cycle arrest (increased acetyl α-tubulin in MCF7)	—	Seifert et al. (2014)
Compound 35, and 39	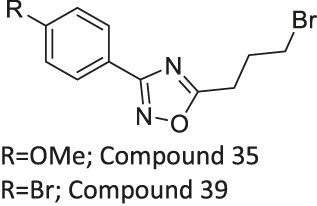	Inhibits SIRT2	- NB4, K562, Karpas299 leukemia, and MDA-MB-231 breast: decreased proliferation (Compound 35)	—	Moniot et al. (2017)
			- NB4, U937, HL-60, OCI-AML3, IMS-M2, OCl-AML2, MV4-11, Kasumi-1, and Karpas299 leukemia cells: decreased proliferation (Compound 39)		
			- NB4 and U937 leukemia cells: increased acetyl α-tubulin (Compound 35 and 39)		
Compound 24a	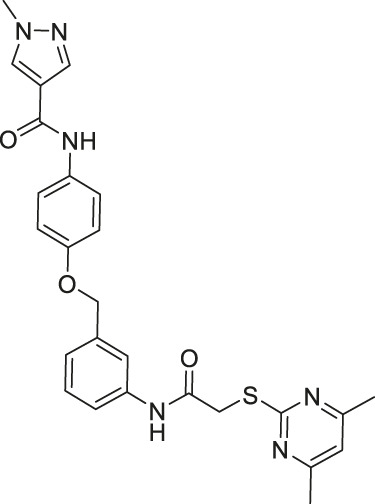	Inhibits SIRT2	- H441 non-small lung: decreased proliferation and migration, and increased acetylated α-tubulin	—	Yang et al. (2018)
TM	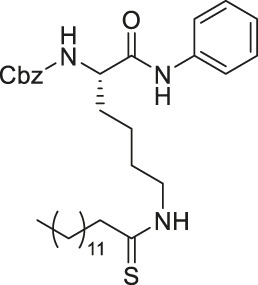	Inhibits SIRT2 with 650-fold selectivity over SIRT1	- MCF7 breast: increased acetyl α-tubulin, decreased proliferation and promoted c-Myc degradation (SIRT2 knockdown decreased proliferation and degraded c-Myc)	- MDA-MB-231 tumor xenograft mice and MMTV-PyMT genetic mice: decreased tumor growths without any toxicity.	(Jing et al., 2016; Spiegelman et al., 2018)
			- MDA-MB-231 and MDA-MB-468: decreased proliferation (SIRT2 knockdown decreased proliferation)		
			- NCI-60 screen: decreased proliferation with GI_50_ less than 10 μM		
			- MCF-10A and HME1 normal breast epithelial: did not alter proliferation		
AF8	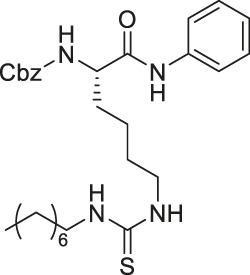	Inhibits SIRT2 with 180-fold selectivity over SIRT1	- HCT-116 colorectal: increased acetyl α-tubulin, and decreased proliferation and colony formation	- HCT-116 colorectal tumor xenograft mice: decreased tumor growth without any toxicity	Farooqi et al. (2019)
			- MCF7, MDA-MB-468, MDA-MB-231 breast, BxPC-3 pancreatic, NCI-H23, A549 lung, and SW948 colorectal: decreased proliferation		
NH4-6/NH4-13	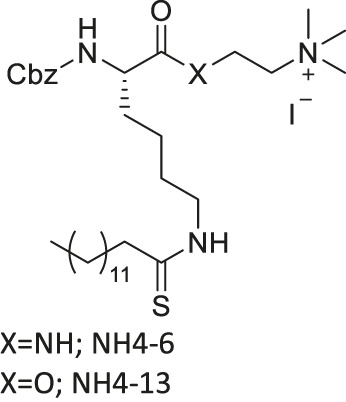	Inhibits SIRT1, SIRT2, and SIRT3 (NH4-6)	- MCF7, MDA-MB-231 breast, HCT-116, SW948 colorectal, HeLa cervical, A549, NCI-H23 lung, MIA-PaCa-2 pancreatic, and U87 glioblastoma: decreased cell proliferation	- HCT-116 colorectal tumor xenograft mice: decreased tumor growth with severe toxicity at high dosage (NH4-6); decreased tumor growth without severe toxicity (NH4-13)	Hong et al. (2021)
		Inhibits SIRT2 with 600-fold selectivity over SIRT1 (NH4-13)			
TM-P4-Thal	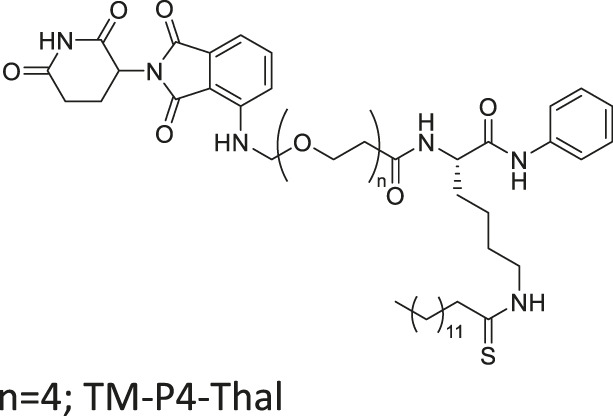	Inhibits SIRT2 with 500-fold selectivity over SIRT1	- MCF7, MDA-MB-231, MDA-MB-468 breast, and BT-549 lung: degraded SIRT2 selectively	—	Hong et al. (2020)
			- MCF7 and MDA-MB-231 breast: decreased proliferation at low concentrations.		
LC-0296	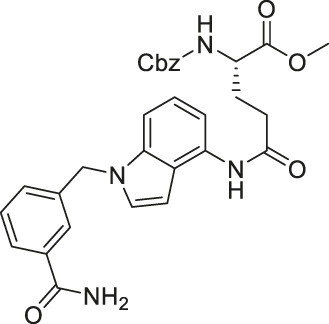	Inhibits SIRT3 with 10-fold selectivity over SIRT2	- UM-SCC-1 and UM-SCC-17B head and neck squamous cell carcinoma (HNSCC): decreased proliferation and enhanced effects of radiation and cisplatin	—	Alhazzazi et al. (2016)
			- UM-SCC-17B (HNSCC): Increased acetylation levels of NDUFA9 and GDH, and ROS levels		
YC8-02	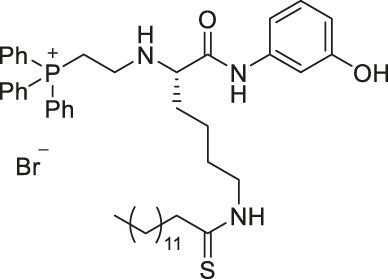	Inhibits SIRT1, SIRT2, and SIRT3	- OCI-LY1 and Karpas422 lymphoma: decreased cell proliferation, increased mitochondrial global acetylation, and decreased TCA cycle metabolites (SIRT3 knockdown had consistent results)	- Karpas422 lymphoma tumor xenograft mice: decreased tumor growth without toxicity (Karpas422 with SIRT3 knockdown also had slower tumor growth)	Li et al. (2019)
			- HBL1, Pfeiffer, SU-DHL4, TMD3, and OCL-LY7 lymphoma: decreased cell proliferation		
DK1-04/DK1-04e	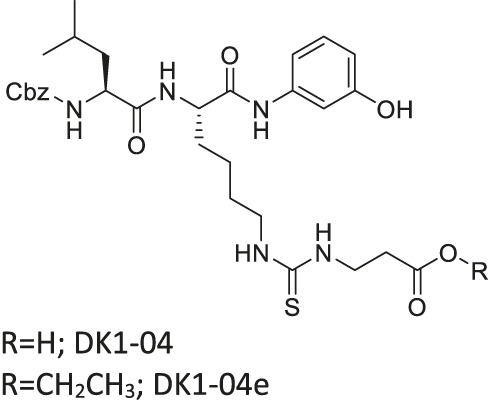	Inhibits SIRT5 selectively (DK1-04)	- MCF7 and MDA-MB-231 breast: (DK1-04e) decreased cell proliferation and colony formation, and increased mitochondrial global succinylation; its inactive derivative (DK1-04e(O) showed weaker cytotoxicity	- MDA-MB-231 breast tumor xenograft mice and MMTV-PyMT genetic mice: decreased tumor growth without toxicity (*Sirt5* deficient PYMT mice impaired tumor growth)	Abril et al. (2021)
			- MDA-MB-231: partial SIRT5 knockout decreased colony formation		
UBCS039	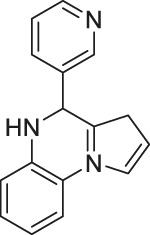	Activates SIRT6	- H1299 non-small cell lung carcinoma: decreased acetyl H3K9 and H3K56, and induced apoptosis (inactive analog UBSC060 did not have any affect)	—	(You et al., 2017; Iachettini et al., 2018)
			- H1299 and HeLa: activated ROS production and increased ATP level (consistent with a previous report on SIRT5 deficiency reducing oxygen consumption and ATP level)		
MDL-800	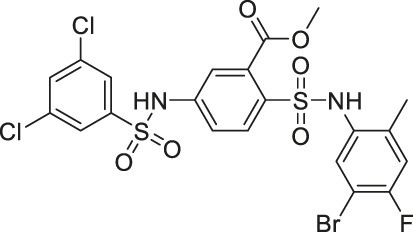	Activates SIRT6	- Bel7405 hepatocellular carcinoma: decreased acetyl H3K9 and H3K56, decreased cell proliferation, and arrested cell cycle (MDL-800 treated SIRT6 knockout did not induce any changes in cell cycle arrest markers)	- Bel7405 hepatocellular tumor xenograft mice and HCC827 non-small lung carcinoma: increased histone H3 acetylation and decreased tumor growth	(Huang et al., 2018; Shang et al., 2021)
			- NCI-60: decreased cell proliferation of the 12 non-small lung carcinoma lines		
			- SIRT6 KO HCC527 and PC9 non-small lung carcinoma: did not affect growth		
Compound 2, 3, and 8	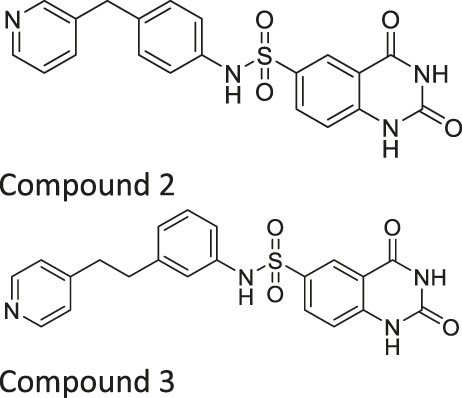 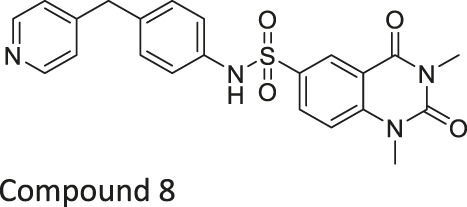	Inhibits SIRT6	- BxPC-3 pancreatic: increased H3K9 acetylation, increased glucose uptake (Compound 3 and 8), decreased proliferation (only Compound 8), and increased antiproliferative affect with gemcitabine (Compound 2 and 3)	—	Sociali et al. (2015)
OSS-128167	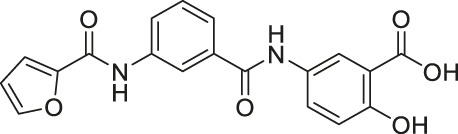	Inhibits SIRT6	- BxPC-3 pancreatic: increased glucose uptake and GLUT-1 expression, and decreased TNF-α	- Mice model of streptozotocin-induced diabetes and high glucose-treated cardiomyocytes: promoted inflammation, oxidative stress, and diabetes-induced cardiomyocyte apoptosis	(Sociali et al., 2015; Huang et al., 2021)
Compound 5 and 11	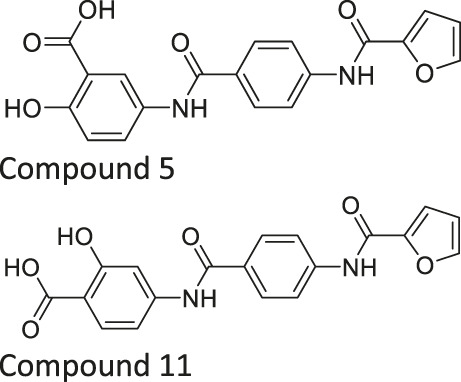	Inhibits SIRT6 with mild inhibition of SIRT2	- BxPC-3 pancreatic: increased H3K9 acetylation, increased glucose uptake, decreased TNF-α, and decreased proliferation with gemcitabine	—	Damonte et al. (2017)
Compound 1	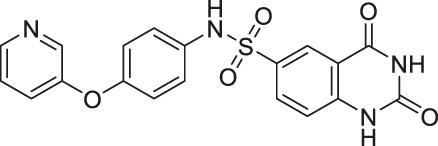	Inhibits SIRT2 and SIRT6	- Dendritic cells: decreased migration	- C57bl/6 mice with MOG35-55 injection: decreased TNFα and neurological impairment, lowered IFNγ and IL12, increased IL10	(Damonte et al., 2017; Sociali et al., 2017; Ferrara et al., 2020)
			- BxPC-3 pancreatic: increased glucose uptake and GLUT-1 expression and decreased TNF-α		
Cambinol	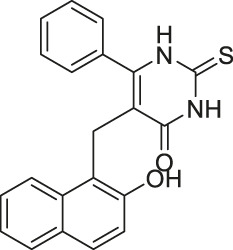	Inhibits SIRT1 and SIRT2	- NCI-H460 lung and HeLa cervical: increased acetylation levels of p53, α-tubulin, FOXO3a, and Ku70	- Orthopedic tumor xenograft mice with HepG2: decreased tumor growth (consistent with SIRT1 knockdown results of intrahepatic xenograft mice study)	(Heltweg et al., 2006; Marshall et al., 2011; Portmann et al., 2013; Ceballos et al., 2021; Lu et al., 2021)
			- RPMI8226 and U266 multiple myeloma: induced apoptosis, cell proliferation impairment, and apoptosis	- TH-MYCN transgenic mice: decreased neuroblastoma formation through N-Myc degradation (SIRT1 knockdown BE (2)-C cells had N-Myc degradation)	
			- HepG2 and Huh7 hepatocarcinoma: decreased cell proliferation, migration, and invasion with sorafenib	—	
Sirtinol	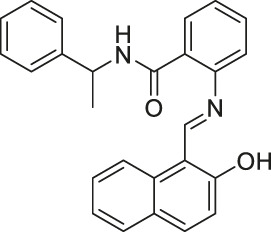	Inhibits SIRT1 and SIRT2	- MCF7 breast and H1299 non-small lung: decreased senescence-like growth and activation of the RAS-MAPK pathway (Similar results with SIRT1 knockdown)	- A549 non-small lung tumor xenograft mice: decreased tumor growth with sodium dichloroacetic acid	(Grozinger et al., 2001; Mai et al., 2005; Ota et al., 2006; Kojima et al., 2008; Kozako et al., 2012; Fong et al., 2014; Zhou et al., 2014; Safari et al., 2017; Ma et al., 2018)
			- H1299 non-small lung and HeLa cervical: decreased cell proliferation	- Subarachnoid hemorrhage rat: lowered SIRT1 expression, damaged the blood-brain barrier and neurological activity, aggravated brain edema, and increased endothelial cell apoptosis	
			- PC3 prostrate, DU145 prostate, S1T adult T-cell leukemia/lymphoma (ATL), and Jurkat ATL: decreased cell proliferation (SIRT1 knockdown decreased proliferation)	- Neonatal rat: decreased cardiacmyocytes	
			- A549 and H1299 non-small lung: decreased cell proliferation with sodium dichloroacetic acid	- Cardiac ischemia preconditioned rats: decreased the infarct size	
Salermide	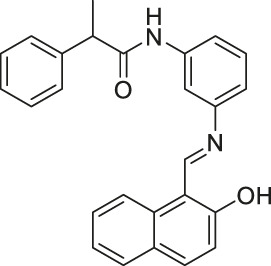	Inhibits SIRT1 and SIRT2	- MOL4 acute lymphomastic leukemia, SW480 colorectal, KG1a acute myelogenous leukemia, and Raji Burkitt’s lymphoma: induced apoptosis through SIRT1 inhibition (confirmed with SIRT1 knockdown)	—	(Lara et al., 2009; Liu et al., 2013)
			- BE(2)-C neuroblastoma and MIA-PaCa-2 pancreatic: decreased cell proliferation through c-Myc and n-Myc degradation (confirmed with SIRT2 knockdown)		
Tenovin-6	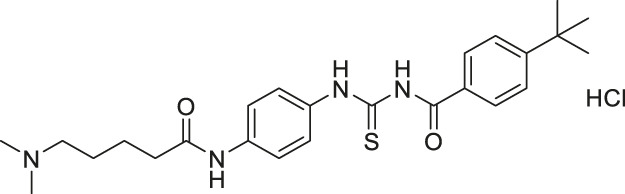	Inhibits SIRT1 and SIRT2, but also target other unknown proteins.	- ARN8 melanoma: decreased cell proliferation	—	(Lain et al., 2008; McCarthy et al., 2012; Dai et al., 2016; Spiegelman et al., 2018; Lee et al., 2019; Igase et al., 2020; Ke et al., 2020)
			- AGS, AGS-EBV, and HGC-27 gastric: decreased cell proliferation and colony formation through increasing acetyl p53 levels		
			- SNU-179, N87, and SNU-1, KATO-III gastric: decreased cell proliferation		
			- HME-1 and MCF-10A normal breast: decreased cell proliferation		
			- A549 non-small lung: decreased proliferation with metformin by HIC1-dependent SIRT1 level reduction		
			- 92.1, Mel-270, Omm-1, Om-2.3 uveal melanoma: decreased migration and proliferation; displayed synergistic effect with Vinblastine		
			- Canine hemangiosarcoma: decreased proliferation with SIRT1-independent mechanism		
			- OCI-Ly1 DLBCL: decreased cell proliferation and induced apoptosis through SIRT1/2/3-independent mechanism		
BZD9L1	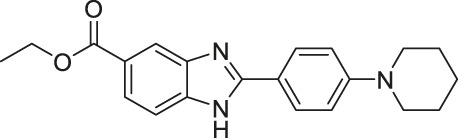	Inhibits SIRT1 and SIRT2	- HCT-116 colorectal, CCRF-CEM leukemia, and MDA-MB-468 breast: decreased cell proliferation	- HCT-116 colorectal tumor xenograft mice: decreased tumor growth with 5-Fluorouracil	(Yoon et al., 2015; Tan et al., 2018; Tan et al., 2019)
			- HCT-116 and HT-29 colorectal: decrsaed cell migration and colony formation		
			- HCT-116 colorectal: increased cell cycle, arrest, and apopotosis, and decreased the spheroid formation with 5-Fluorouracil		
Compound 18	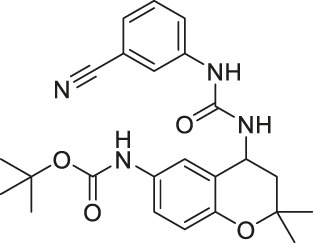	Inhibits SIRT1 and SIRT2	- HS683 and U373 glioma: decreased proliferation (consistent with SIRT1/2 knockdown) and increased acetylation of H4, H3K56 and α-tubulin	- HS683 an dU373 glioma zebrafish xenotransplant model: decreased tumor growth	Schnekenburger et al. (2017)
			- NCI-60 screen: decreased proliferation with an average GI_50_ of 3 μM		
Compound 3g	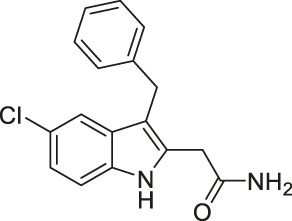	Inhibits SIRT1 and SIRT2	- K562 leukemia, HCT-116, HT-29 colorectal, H460, A549 lung, and MCF7 breast: decreased proliferation (need additional data to show cellular inhibition of SIRT1 and 2)	—	Laaroussi et al. (2020)
MC2494	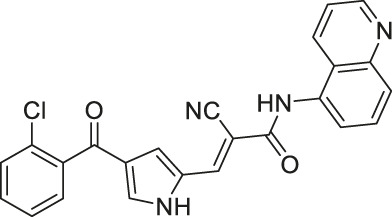	Inhibits SIRT1, SIRT2, and SIRT3	- U937 lymphoma: decreased metabolic activity and proliferation; lowered decreased ATP production and expression level of PGC1α and PGC1β	—	(Carafa et al., 2018; Carafa et al., 2020)
JH-T4	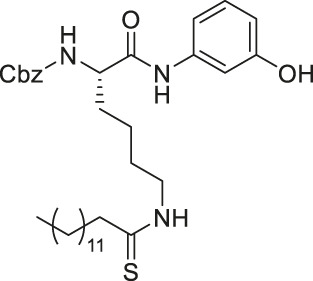	Inhibits SIRT1, SIRT2, and SIRT3	- MCF7, MDA-MB-231 breast, HCT-116 colorectal, NCI-H23 lung, HME1 and MCF-10A normal epithelial cells: decreased cell proliferation	—	Spiegelman et al. (2019)
35. Splitomicin	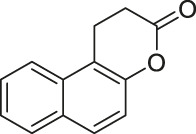	Inhibits yeast sirtuins	- Human endothelial cells: increased and activated tissue factor expression (confirmed with SIRT1 knockdown)	- Photochemical injury mice: promoted carotid artery thrombus formation	Breitenstein et al. (2011)

**TABLE 2:** Cell lines affected by SIRT1 Inhibitors.

Type	Line	SIRT1 Inhibitor
		EX-527
Glioma	U87MG	↓ proliferation
	LN0299	↓ proliferation
Bladder	5637	↓ proliferation
	T24	↓ proliferation
Lung	H-460-R- cisplatin resistant	↑ senstivity to cisplatin
	A549	↑senstivity with MK-1775
	A549 (xenograft)	↓ tumor growth with MK-1775
Endometrial carcinoma	HEC151	↓ proliferation
	HEC1B	↓ proliferation
	HHUA	↓ proliferation
	HHUA (xenograft)	↓ tumor growth
Prostate	PC-3	↑ senstivity to vesicular stomatitis virus oncolysis
Pancreatic	PANC-1	↓ proliferation ↑senstivitiy to gemcitabine
	PANC-1 (xenograft)	↑ tumor growth
Lymphoma	Chemo-resistant K562	↑ sensitivity to 17-AAG and AUY922

**TABLE 3:** Cell lines affected by SIRT2 Inhibitors (#1 set).

Type	Line	SIRT2 Inhibitor						
		AGK2	AK7	SirReal2	RK-91230156	NCO-90/141	NPD11033	Compound 6f/12a
Glioma	GB2	↓ proliferation	—	—	—	—	—	—
	GB2 (xenograft)	—	↓ tumor growth	—	—	—	—	—
	GB3	↓ proliferation	—	—	—	—	—	—
	GB11	↓ proliferation	—	—	—	—	—	—
	GB16	↓ proliferation	—	—	—	—	—	—
Lung	A549	—	—	↓ proliferation	—	—	—	↓ proliferation
	H520	—	—	↓ proliferation	—	—	—	—
Pancreatic	PANC-1	—	—	—	—	—	↓ proliferation	—
Colorectal	HCT-116	↓ effect of chemotherapeutic agents	—	↓ proliferation	—	—	—	—
	SW620	↑ effect of chemotherapeutic agents	—	—	—	—	—	—
	SW948	—	—	↓ proliferation	—	—	—	—
Leukemia	S1T	—	—	—	—	↑ acetylation of H4	—	—
	MT-2	—	—	—	—	↑ acetylation of H4	—	—
	Jurkat	—	—	—	—	↑ acetylation of H4	—	—
	HL60	—	—	—	—	↑ acetylation of H4	—	—
Lymphoma	Chemo-resistant K562	—	—	↓ proliferation	—	—	—	—
	K562	—	—	↓ proliferation	—	—	—	—
Gastric	HGC-27	—	—	↓ proliferation	—	—	—	—
	MGC-803	—	—	↓ proliferation	—	—	—	—
Breast	MCF7	—	—	↓ proliferation	↓ proliferation	—	—	↓ proliferation
	MDA-MB-231	—	—	↓ proliferation	—	—	—	—
	MDA-MB-468	—	—	↓ proliferation	—	—	—	—
Cervical	HeLa	—	—	↓ proliferation	—	—	—	—

**TABLE 4:** Cell lines affected by SIRT2 Inhibitors (#2 set).

Type	Line	SIRT2 Inhibitor							
		Compound 35	Compound 39	Compound 24a	Compound 53	TM	AF8	NH4-13	TM-P4-Thal
Various	NCI-60	—	—	—	—	↓ proliferation	—	—	—
Glioma	U87MG	—	—	—	—	—	—	↓ proliferation	—
	Lung	A549	—	—	—	—	—	↓ proliferation	↓ proliferation	—
		NCI-H23	—	—	—	—	—	—	↓ proliferation	—
	Non-small Lung	H441	—	—	↓ proliferation	—	—	—	—	—
	Pancreatic	BxPC-3	—	—	—	—	—	↓ proliferation	—	—
		Mia-PaCa-2	—	—	—	—	—	—	↓ proliferation	—
Colorectal	HCT-116	—	—	—	—	—	↓ proliferation	↓ proliferation	—
	HCT-116 (xenograft)	—	—	—	—	—	↓ tumor growth	↓ tumor growth	—
	SW948	—	—	—	—	—	↓ proliferation	↓ proliferation	—
Leukemia	HL60	—	↓ proliferation	—	—	—	—	—	—
	NB4	↓ proliferation	↓ proliferation	—	—	—	—	—	—
Lymphoma
	K562	↓ proliferation	—	—	—	—	—	—	—
	Karpas299	↓ proliferation	↓ proliferation	—	—	—	—	—	—
	U937	—	↓ proliferation	—	—	—	—	—	—
	OCI-AML3	—	—	—	—	—	—	—	—
	IMS-M2	—	↓ proliferation	—	—	—	—	—	—
	OCI-AML3	—	↓ proliferation	—	—	—	—	—	—
	MV4-11	—	↓ proliferation	—	—	—	—	—	—
	Kasumi1	—	↓ proliferation	—	—	—	—	—	—
Breast	MCF7	—	—	—	↓ proliferation	↓ proliferation	↓ proliferation	↓ proliferation	↓ proliferation
	MDA-MB-231	↓ proliferation	—	—	—	↓ proliferation	↓ proliferation	↓ proliferation	↓ proliferation
	MDA-MB-231 (xenograft)	—	—	—	—	↓ tumor growth	—	—	—
	MDA-MB-468	—	—	—	—	↓ proliferation	↓ proliferation	—	—
Cervical	HeLa	—	—	—	—	—	—	↓ proliferation	—
Normal Epithelial	MCF-10A	—	—	—	—	No effect	—	—	—
	HME1	—	—	—	—	No effect	—	—	—

**TABLE 5:** Cell lines affected by SIRT3/SIRT5 inhibitors.

		SIRT3 Inhibitor		SIRT5 Inhibitor
Type	Line	LC-0296	YC8-02	DK1-04e
Lymphoma	OCL-LY-1	—	↓ proliferation	—
	Karpas422	—	↓ proliferation	—
	Karpas422 (xenograft)	—	↓ tumor growth	—
	HBL1	—	↓ proliferation	—
	Pfeiffer	—	↓ proliferation	—
	SU-DHL4	—	↓ proliferation	—
	TMD3	—	↓ proliferation	—
	OCL-LY7	—	↓ proliferation	—
Breast	MCF7	—	—	↓ proliferation
	MDA-MB-231	—	—	↓ proliferation
	MDA-MB-231 (xenograft)	—	—	↓ tumor growth
HNSCC	UM-SCC-1	↓ proliferation	—	—
	UM-SCC-17B	↓ proliferation	—	—

**TABLE 6:** Cell lines affected by SIRT6 modulators.

		SIRT6 Activator		SIRT6 Inhibitor				
Type	Line	UBCS039	MDL-800	Compound 2	Compound 3	Compound 8	Compound 5	Compound 11
NCI-60	NCI-60	—	↓ proliferation	—	—	—	—	—
Non-small Lung	H1299	↓ proliferation	—	—	—	—	—	—
Hepatocellular carcinoma	Bel7405	—	↓ proliferation	—	—	—	—	—
Pancreatic	BxPC-3	—	—	↑ senstivity to gemcitabine	↑ senstivity to gemcitabine	↓ proliferation	↑ senstivity to gemcitabine	↑ senstivity to gemcitabine

## Overview of Sirtuin Modulators

Numerous activators and inhibitors of sirtuins have been synthesized. Before discussing the evaluation of the sirtuin modulators in the disease models, we will briefly provide an overview of the sirtuin modulators and their efficiency. The structures and other information of these modulators are summarized in Table 1.

There are several compounds that regulates the cellular level of NAD^+^ and consequently modulate sirtuins. Adding precursors of NAD^+^ like nicotinamide riboside (NR) or nicotinamide mononucleotide (NMN) to the cells increased the overall NAD^+^ level, thereby activating sirtuins (Bonkowski and Sinclair, 2016; Trammell et al., 2016). Furthermore, inhibiting CD38, which converts NAD^+^ to various products, with apigenin or quercetin activated sirtuins (Aksoy et al., 2006; Lee, 2006; Escande et al., 2013). Because this review specifically summarizes modulators that directly bind to sirtuins, we will not explain these indirect modulators in detail.

### SIRT1 Modulators

Because SIRT1 was initially connected to longevity, many small-molecule activators have been developed and tested for anti-aging purposes (Howitz et al., 2003; Borra et al., 2005; Milne et al., 2007). However, many follow-up studies questioned the activating mechanism, as the activation was only observed when using aminomethylcoumarin or fluorophore-tagged peptide substrate for *in vitro* assays (Beher et al., 2009; Pacholec et al., 2010). The original authors have rebutted this by showing direct allosteric SIRT1 activation with biochemical assays and crystallography structures (Dai et al., 2010; Hubbard et al., 2013). Later, it was found that a SIRT1 activator could also inhibit SIRT3 (Nguyen et al., 2013). Thus, the effects of SIRT1 activators may not necessarily come from the activated SIRT1. Lastly, there are already numerous review articles covering these SIRT1 activators (Hubbard and Sinclair, 2014; Dai et al., 2018; Fujita and Yamashita, 2018; Salehi et al., 2018; Iside et al., 2020). Due to these reasons, we have decided to leave out the discussion on SIRT1 activators in this review.

For SIRT1, EX-527 or selistat is the most used SIRT1 selective inhibitor in biological studies. EX-527 inhibited SIRT1 with an IC_50_ of 38 nM with 200-fold selectivity over SIRT2 and SIRT3 (Solomon et al., 2006). When using a H3K56 fluorogenic substrate, 200 μM of EX-527 showed 56% SIRT6 inhibition (Kokkonen et al., 2014). Nevertheless, EX-527 still showed significantly stronger SIRT1 selectivity over SIRT6. In cells, EX-527 treatment significantly increased the acetylation of p53, a SIRT1 deacetylation substrate (Solomon et al., 2006). Several other SIRT1 inhibitors were reported, but whether they selectively inhibit SIRT1 was not validated (Kozako et al., 2015; Ghosh et al., 2017).

### SIRT2 Inhibitors

AGK2 was synthesized from a high-throughput screening and showed SIRT2 inhibition with an IC_50_ of 8 μM and 5-fold selectivity for SIRT2 over SIRT1 (Outeiro et al., 2007). In cells, AGK2 significantly increased acetylation levels of α-tubulin, a SIRT2 deacetylation substrate (Mangas-Sanjuan et al., 2015; Spiegelman et al., 2018). A limitation of using AGK2 in biological studies is its poor solubility in water and ethanol. Furthermore, according to SelleckChem, its maximum solubility in DMSO is only about 23 mM at 50°C. The poor solubility could make it difficult to use it in cells and animals.

AK-7 inhibited SIRT2 with an IC_50_ of 15.5 μM and does not inhibit SIRT1 or SIRT3 (Taylor et al., 2011). The main advantage of AK-7 is its brain permeability. After 2 h of intraperitoneal injection to mice, about 2 μM of AK-7 was detected in the brain. As such, utilizing AK-7 in neurological diseases could be helpful.

SirReal2 binds a hydrophobic pocket of SIRT2, where the long-chain fatty acyl group of substrates occupies (Rumpf et al., 2015). SirReal2 inhibited SIRT2 with an IC_50_ of 140 nM without any inhibition of other sirtuins. In cells, SirReal2 led to increase in acetylation levels of α-tubulin and BuBR1, acetylation targets of SIRT2. Furthermore, SirReal2 did not affect acetylation level of p53, a substrate of SIRT1 (Rumpf et al., 2015; Spiegelman et al., 2018).

From a large library screening of 140,000 compounds, RK-91230156 was discovered to inhibit SIRT2 over SIRT1, SIRT3, HDAC1, and HDAC6. Moreover, RK-91230156 inhibits SIRT2 with an IC_50_ of 0.18 μM. RK-91230156 significantly increased acetylation of eIF5a, another reported SIRT2 deacetylation target (Shah et al., 2016).

NCO-90 and NCO-141 are nicotinamide-derived SIRT2 selective inhibitors with IC_50_ values of 1 and 0.57 μM, respectively. In HCT-116 colorectal cells, treatment with NCO-90 increased the acetylation level of α-tubulin, while did not affect the acetylation level of p53 (Suzuki et al., 2012). By attaching NCO-90 to a thioacylated lysine, KPM-2 was synthesized. Unlike NCO-90, KPM-2 simultaneously inhibits SIRT1, SIRT2, and SIRT3. Its IC_50_ values for SIRT1, SIRT2, and SIRT3 were 1.56, 0.055, and 9.49 μM, respectively. In cells, KPM-2 dose-dependently increased the acetylation of α-tubulin (Mellini et al., 2017). Compound 53 is an NCO-90-based diketopiperazine compound that inhibits SIRT2 by concurrently occupying the selectivity pocket, substrate-binding site, and NAD^+^ binding site. Compound 53 inhibited SIRT2 at an IC_50_ of 0.31 μM with 250 and 223-fold selectivity over SIRT1 and SIRT3, respectively. In MCF7 cells, Compound 53 increased the acetylation level of α-tubulin (Mellini et al., 2019).

Discovered from high-throughput screening of RIKEN NPDepo chemical library, NPD11033 selectively inhibited SIRT2 deacetylase activity with IC_50_ of 0.46 μM, but did not inhibit SIRT2 defatty-acylase activity (Kudo et al., 2018). NPD11033 increased acetylation level of eIF5A in PANC-1 pancreatic cells (Kudo et al., 2018).

Compound 6f and Compound 12a are Chroman-4-one and chromone-based SIRT2 inhibitors with IC_50_ of 3.7 and 12.2 μM, respectively. They increased the acetylation level of α-tubulin in cells (Seifert et al., 2014). 1,2,4-oxadizazole-based Compound 35 and 39 inhibited SIRT2 through an uncompetitive mechanism against α-tubulin peptide substrate and NAD^+^. Their SIRT2 IC_50_ were 10.4 and 1.5 μM, respectively. In NB4 and U937 cells, both Compound 35 and 39 increased acetyl α-tubulin (Moniot et al., 2017). A SIRT2 selective inhibitor, Compound 24a was discovered from a SAR study of N-(3-(phenoxymethyl)phenyl)acetamide derivatives (Yang et al., 2018). It binds to the hydrophobic acyl pocket and inhibits SIRT2 with an IC_50_ of 0.815 μM. Furthermore, Compound 24a did not inhibit other sirtuins at 100 μM. In H441 non-small lung cancer cells, Compound 24a increased the acetylation level of α-tubulin (Yang et al., 2018).

As mentioned, sirtuins form a covalent O-acyl-ADP-ribose intermediate during its catalytic reaction. Many thioacyl lysine compounds (or the corresponding thiourea) form similar covalent intermediates, but the substitution of the oxygen atom by sulfur inhibits the downstream decomposition of the intermediate, which occupies the active site and inhibits sirtuins (Smith and Denu, 2007; Feldman et al., 2015; Jing et al., 2016).

TM contains a thiomyristoyl lysine with an N-terminal carboxybenzoyl (Cbz) group and a C-terminal amide formed with aniline (Jing et al., 2016). TM selectively inhibited SIRT2 by forming a stalled covalent intermediate, which was captured by mass spectrometry. In cells, treatment of TM increased acetylation of α-tubulin in a dose-dependent manner. Moreover, its SIRT2 IC_50_ was 0.04 μM with 650-fold selectivity over SIRT1. TM could not inhibit SIRT3 even at 50 μM (Spiegelman et al., 2018). AF8, a derivative of TM with a thiourea moiety mimicking the thioacyl group, also formed a stalled covalent intermediate to selectively inhibit SIRT2 with an IC_50_ of 0.061 μM and 180-fold selectivity over SIRT1. In HCT-116 colorectal cancer cells, AF8 increased the acetylation of α-tubulin in a dose-dependent manner but did not change the acetylation of p53 (Farooqi et al., 2019). Two other TM derivatives, NH4-6 and NH4-13 containing a trimethylammonium moiety (Hong et al., 2021), have excellent aqueous solubility compared to TM. The difference between the two inhibitors is that NH4-6 has an amide linkage and NH4-13 has an ester linkage between the lysine and the trimethylammonium moiety. This small difference led to a completely different inhibition profile. NH4-6 with the amide bond simultaneously inhibits SIRT1, 2, and 3 with IC_50_ of 3, 0.032, and 2.3 μM, respectively. Meanwhile, NH4-13 with the ester bond selectively inhibits SIRT2 with an IC_50_ of 0.087 μM. Furthermore, in cells, NH4-6 increased acetylation levels of p53, α-tubulin, and IDH2, acetylation targets of SIRT1, SIRT2, and SIRT3, respectively. Meanwhile, NH4-13 increased the acetylation levels of alpha-tubulin, but not of p53 and IDH2 (Hong et al., 2021).

Many reported SIRT2 inhibitors, including TM, efficiently inhibit SIRT2’s deacetylase activity, but not its defatty-acylase activity. However, converting them to proteolysis-targeting chimeras (PROTAC) could enable them to inhibit both activities. TM-P4-Thal is a PROTAC SIRT2 inhibitor with thalidomide on one end and TM on the other end, connected by a polyethylene glycol (PEG) linker. The thalidomide recruits CRBN E3 ligase, while TM interacts with SIRT2. Such recruitment leads to polyubiquitination of SIRT2, thereby inducing proteolysis-mediated degradation. Degradation of SIRT2 could eradicate both SIRT2 activities in cells. As such, TM-P4-Thal had increased acetylation level of α-tubulin and fatty acylation level of K-Ras4a, a defatty-acylation substrate of SIRT2 (Hong et al., 2020).

### SIRT3 Inhibitors

Several SIRT3 inhibitors that have been reported. LC-0296 based on glutamic acid with heterocyclic rings inhibited SIRT3 with an IC_50_ of 3.6 μM and a 10-fold selectivity over SIRT2 (Alhazzazi et al., 2016). LC-0296 increased mitochondrial global acetylation and several SIRT3-specific deacetylation targets, including NDUFA9 and GDH (Alhazzazi et al., 2016).

YC8-02 is another mechanism-based sirtuin inhibitor based on TM, with 3-aminophenol replacing the aniline, and triphenylphosphine group replacing the Cbz group of TM (Li et al., 2019). With the additional hydroxyl group of 3-aminophenol, YC8-02 simultaneously inhibits SIRT1, SIRT2, and SIRT3 with IC_50_ of 2.8, 0.062, and 0.53 μM, respectively. Because SIRT3 is localized in mitochondria, the triphenylphosphine group, known as a mitochondrial targeting moiety, helps to direct the inhibitor to the mitochondria and thus increase SIRT3 targeting in cells. After treating DLBLC cells, YC8-02 was detected by mass spectrometry in the purified mitochondria extract, which confirms the mitochondrial targeting of YC8-02. In addition, increased global mitochondrial acetylation was observed upon treatment of YC8-02. Similar treatment with JH-T4, which also inhibits SIRT1, SIRT2, and SIRT3 *in vitro* but does not possess the triphenylphosphine group, did not alter the acetylation level of mitochondria.

### SIRT5 Inhibitors

Several SIRT5 inhibitors have been developed, but only a few of them were tested in cellular and mice models. A dipeptide SIRT5 selective inhibitor, DK1-04 contains a lysine with thiourea moiety mimicking the glutaryl group (Abril et al., 2021). It inhibits SIRT5 by forming a covalent intermediate with NAD^+^. DK1-04 selectively inhibited SIRT5 with an IC50 of 0.34 μM. Because the carboxylic acid of DK1-04 hinders the cell permeability, DK1-04e, a pro-drug with an ethyl ester group on the carboxylic acid, was synthesized for biological evaluations. In cells, DK1-04e had increased mitochondrial lysine succinylation, which validates its cellular SIRT5 inhibition.

### SIRT6 Modulators

Many SIRT6 activators have been recently developed and tested in cancer studies. Pyrrolo[1,2-α]quinoxaline-derived UBCS039 activated SIRT6 at EC_50_ of 38 μM by binding to its fatty acyl pocket (You et al., 2017). UBCS039 also mildly (2-fold) activated SIRT5 at 100 μM, but not SIRT1, SIRT2, and SIRT3. In an *in vitro* SIRT6 deacetylation reaction using full-length histones or nucleosome from HeLa cells, UBCS039 significantly enhanced the deacetylation of H3K18. In H1299 non-small cell lung carcinoma cells, treatment of UBSC039 decreased acetylation of histone H3 K9 and K56, two known SIRT6 deacetylation targets (Iachettini et al., 2018).

Utilizing the Allosite server, MDL-800 was discovered to activate SIRT6 by binding a pocket around Phe83 and Phe86 residues of SIRT6. MDL-800 activated SIRT6 with an EC_50_ of 10.3 μM and 10-fold selectivity over SIRT2, SIRT5, and SIRT7. Furthermore, MDL-800 did not activate SIRT1, SIRT3, SIRT4, or HDAC1-11. In BEL7405 hepatocellular carcinoma cells, MDL-800 decreased the acetylation level of H3K9 and H3K56, which are known SIRT6 deacetylation targets (Huang et al., 2018).

In addition to the activators, several SIRT6 selective inhibitors have been reported although they are not very potent. Quinazolinedione-based Compound 2, 3, and 8 inhibited SIRT6 at IC_50_ of 60, 37, and 49 μM, respectively. Compound 2 mildly inhibited SIRT1 and SIRT2 with IC_50_ of 238 and 159 μM, respectively. Also, Compound 3 mildly inhibited SIRT2 with an IC_50_ of 85 μM. Compound 8 was 5-fold selective for SIRT6 over SIRT2. Compound 3 and 8 showed 11 and 133-fold SIRT6 selectivity over SIRT1. In BxPC-3 pancreatic cells, treatment of Compound 2, 3, and 8 increased acetylation of H3K9 (Sociali et al., 2015).

Salicylate-based OSS-128167 inhibited SIRT6 with an IC_50_ of 89 μM and 17 and 8-fold selectivity over SIRT1 and SIRT2, respectively. In BxPC-3 pancreatic cells, OSS-128167 increased the acetylation level of H3K9 and glucose uptake, and decreased TNF-α secretion. Similar effects were also observed when SIRT6 was knocked down (Parenti et al., 2014). Later, Compound 5 and 11 were designed to have stronger potency than OSS-128167. These inhibitors had inhibited SIRT6 with IC_50_ of 34 and 22 μM by binding to the nicotinamide and substrate pocket. Furthermore, both inhibitors showed 14-fold selective SIRT6 inhibition over SIRT1 and SIRT2. In BxPC-3 pancreatic cells, Compound 5 and 11 increased the acetylation level of H3K9 (Damonte et al., 2017). In this same study, Compound 1 was also reported to inhibit SIRT6 with an IC_50_ of 106 μM. Additionally, Compound 1 inhibited SIRT2 at an IC_50_ of 114 μM (Damonte et al., 2017). Although Compound 1 inhibited both SIRT2 and SIRT6, Compound 1 had the most suitable physicochemical properties for the *in vivo* studies, as it had moderate oral absorption, aqueous solubility, and metabolic stability (Sociali et al., 2017).

### Pan-Sirtuin Inhibitors

Because sirtuins share similar structures, many modulators simultaneously interact with multiple sirtuins.

Identified in 2006, Cambinol moderately inhibits SIRT1 and SIRT2 through competitive inhibition against histone H4 and noncompetitive inhibition against NAD^+^. Cambinol inhibited SIRT1 and SIRT2 at IC_50_ of 56 and 59 μM, respectively. In NCI H460 cells, treatment of Cambinol had significantly increased acetylation of p53 and α-tubulin (Heltweg et al., 2006).

Through high-throughput screening and optimization, Sirtinol was identified as a SIRT1 and SIRT2 inhibitor, with IC_50_ of 131 and 58 μM, respectively (Grozinger et al., 2001; Mai et al., 2005). However, it failed to increase global acetylation levels of histone and α-tubulin in cells (Grozinger et al., 2001). Salermide contains a reversed amide structure of sirtinol and shows mild SIRT1 and SIRT2 inhibitions. Salermide showed 80% inhibition of SIRT1 and SIRT2 at 100 and 25 μM, respectively. Only in some specific cell lines, Salermide treatment increased the acetylation of α-tubulin and p53. Also, it did not affect global H4 acetylation levels, a previously reported SIRT2 substrate (Lara et al., 2009). Thus, when using Sirtinol and Salermide, additional confirmation will be needed to verify whether the observed effects are due to the sirtuin inhibition.

From screening compounds for p53 activation, Tenovin-6 was discovered to simultaneously inhibit SIRT1 and SIRT2 with IC_50_ of 26 and 9 μM, respectively (Lain et al., 2008; McCarthy et al., 2012). In ARN8 melanoma cells, Tenovin-6 increased the acetylation level of p53 and α-tubulin. The increase of acetylation level of α-tubulin by Tenovin-6 was rescued with SIRT2 overexpression (Lain et al., 2008). Despite its effective SIRT1 and SIRT2 inhibition, Tenovin-6 may target other proteins in cells. For instance, Tenovin-6 impaired cellular growth of canine hemangiosarcoma cells through a SIRT1-independent mechanism (Igase et al., 2020). Tenovin-6’s effect in DLBCL cells is also thought to be SIRT1/2/3 independent (Yuan et al., 2017). Another drawback of Tenovin-6 was its over-toxicity. In a direct comparative study with other SIRT2 inhibitors, even though Tenovin-6 showed the strongest antiproliferative effect, it also killed tested normal epithelial cell lines (Spiegelman et al., 2018). Thus, when using Tenovin-6 *in vitro* or *in vivo*, extra care is need to rule out off-target effect and avoid toxicity issues.

BZD9L1, a highly fluorescent sirtuin inhibitor, inhibited SIRT1 and SIRT2 with IC_50_ of 42.9 and 9 μM, respectively. Based on the docking study with SIRT2, BZD9L1 occupied where adenosine diphosphate ribose bound. In HCT-116 colorectal cells, BZD9L1 increased acetylation of p53 after etoposide-induced DNA damage and α-tubulin. Because BZD9L1 possesses intrinsic fluorescence, the cellular distribution of BZD9L1 in HCT-116 and CCD18 colon fibroblasts could be detected using fluorescence microscopy (Yoon et al., 2015).

N-aryl-N’-3,4-dihydro-2,2-dimethyl-2H-1-benzopyran-4-yl)ureas-derived Compound 18 simultaneously inhibited SIRT1 and SIRT2 with IC_50_ of 6.2 and 4.2, respectively (Schnekenburger et al., 2017). In U373 and Hs683 glioblastoma, treatment of Compound 18 increased acetylation of histone H4 and α-tubulin (Schnekenburger et al., 2017).

Compound 3g, an achiral indole analog of EX-527, showed potent inhibition against both SIRT1 and SIRT2 with IC_50_ of 4.9 and 1 (0.62–1.4) μM, respectively (Laaroussi et al., 2020).

MC2494 inhibited all SIRT1-6 with IC_50_ values of 38.5 and 58.6 μM for SIRT1 and SIRT2. Upon thermal stress, MC2494 protected SIRT1, SIRT2 and SIRT3 against degradation. In cells, MC2494 increased not only the global lysine acetylation but also acetylation levels of p53, tubulin, histone H3, and histone H4 (Carafa et al., 2018).

JH-T4 is an analog of TM with a 3-aminophenol group replacing the aniline part of TM (Spiegelman et al., 2019). Interestingly, with just one additional hydroxyl group, JH-T4 inhibits SIRT1, SIRT2, and SIRT3. From the docking study with SIRT2, the hydroxyl group forms a hydrogen bond interaction with the protein backbone of the sirtuins, which could have contributed to its simultaneous inhibition. Moreover, JH-T4 inhibits both SIRT2 deacetylase and defatty-acylase, as increased acetylation of α-tubulin and fatty-acylation of K-Ras4a were observed upon treatment of JH-T4 (Spiegelman et al., 2019).

## Sirtuin Modulators in Cancer

Because sirtuins are involved in a plethora of biological pathways, they could play both tumor suppressor and activator roles (Bosch-Presegue and Vaquero, 2011; Hu et al., 2014). In this section, we will briefly highlight the roles of sirtuins and their modulators in tumorigenesis.

### SIRT1 Inhibitors in Cancer

SIRT1 could serve as a tumor suppressor as it deacetylates and inactivates various tumor-promoting transcriptional factors. For instance, SIRT1 deacetylates K310 of NF-κB and attenuates its transcriptional activity, which consequently suppress inflammation and turmorigenesis (Chen et al., 2002; Yeung et al., 2004). This promotes TNF-α induced apoptosis. Also, SIRT1 deacetylates and inactivates HIF-1α, which leads to repression of HIF-1α target genes. In mice, xenografted HT1080 tumors with SIRT1 overexpression formed smaller tumors than the xenografted wild-type HT1080 tumors (Lim et al., 2010). Knockdown of SIRT1 in HMLER breast cancer cells increased metastasis. In the same study, SIRT1 was reported to deacetylate Smad4 and subsequently keep β-catenin interacting with E-adherin. This would suppress the epithelial-to-mesenchymal transition (Simic et al., 2013).

In contrast, some studies reported SIRT1 as a tumor activator. SIRT1 deacetylates FOXO1 and inhibits FOXO1-induced apoptosis (Yang et al., 2005). SIRT1 overexpression increases the expression of c-Myc, a key oncoprotein that increases the expression of many tumor proliferating genes. Furthermore, SIRT1 deacetylates c-Myc to promote its transcriptional activity (Menssen et al., 2012). SIRT1 deacetylates and represses p53, which exerts antiproliferative effects, including growth arrest, apoptosis, and cell senescence. Deacetylation of p53 also translocates p53 to mitochondria, which suppresses its transcriptional activity (Han et al., 2008). In MCF7 breast cancer cells, overexpression of SIRT1 increased the proliferation, migration, and motility by increasing the POLD1 expression (Xu et al., 2018). The dual role of SIRT1 is also depicted in HCT-116 colorectal cells. Heterozygous deletion of SIRT1 increased c-Myc expression, and thereby promoted tumor growth. Meanwhile, homozygous deletion of SIRT1 promoted apoptosis and delayed cancer formation (Ren et al., 2017). Thus, the role of SIRT1 in cancer may vary depending on the context.

There are only a few reports of EX-527 producing effective anticancer effects as a single agent (see summary in Table 2). In U87MG and LN-299 glioma cells, EX-527 decreased the cellular proliferation and anchorage-independent colony formation through p53 and acetylated-p53 upregulation, and caspase-dependent apoptosis activation (Wang et al., 2020). In 5637 and T24 bladder cancer cells, SIRT1 overexpression promoted cell proliferation and GLUT1 expression. Hence, treatment of EX-527 in these cells had an opposite effect, which decreased the proliferation, glycolysis, and glucose uptake (Chen et al., 2019).

In contrast, many reports indicate that EX-527 can enhance and synergize with other treatments. For instance, by inhibiting the deacetylation of XRCC1, EX-527 increased sensitivity of H460-R cisplatin-resistant lung cancer cells to cisplatin. Overexpression of SIRT1 rescued such effect, while the knockdown of SIRT1 also made cells vulnerable to cisplatin (Yousafzai et al., 2019). In addition, EX-527 impaired the proliferation of several cisplatin-resistant endometrial carcinoma cells, such as HEC151, HEC1B, and HHUA. In HHUA cells, overexpression of SIRT1 reversed enhanced cisplatin resistance. In the HHUA tumor xenograft mice model, treatment with EX-527 significantly detained the tumor growth (Asaka et al., 2015). In PC-3 prostate cancer, SIRT1 knockdown or EX-527 increased the effect of vesicular stomatitis virus oncolysis treatment (Muscolini et al., 2019). In chemo-resistant stem-like cells from leukemia K562, EX-527 or SIRT1 knockdown increased the effect of Hsp90 inhibitors like 17-AAG and AUY922. SIRT1 inhibition or depletion decreased expression of heat shock proteins, and consequently increased the effects of Hsp90 inhibitors (Kim et al., 2015). EX-527 and SIRT1 knockdown also induced a synergistic anticancer effect with MK-1775, a WEE1 inhibitor. In both cellular and xenograft mice models, treatment of EX-527 and MK-1775 suppressed the growth of A549 lung cancer cells. Meanwhile, a single treatment of EX-527 or MK-1775 did not affect the growth. Mechanistically, SIRT1 can deacetylate and inhibit NBS1 and Rad51 in homologous recombination repair. Thus, the combination of EX-527 and MK-1775 induced complete damage in the DNA replication process (Chen et al., 2017). Lastly, in PANC-1 pancreatic cancer cells, EX-527 itself decreased proliferation, and synergistically increased the antiproliferative effect of gemcitabine. However, in PANC-1 tumor xenograft mice, EX-527 promoted tumor growth and did not show any additive or synergistic effect with gemcitabine (Oon et al., 2015).

Based on all the data, it is likely that SIRT1’s major role is to help cells survive various stresses. Depending on the nature and level of the stresses in specific cancer cells, inhibiting SIRT1 may produce pro-tumor or anti-tumor activity. This could also explain why SIRT1 inhibition can synergize with other small molecules.

### SIRT2 Inhibitors in Cancer

Although SIRT2 was initially reported to be a tumor suppressor, as *Sirt2* knockout mice developed more tumors than wild-type mice as they age (Kim et al., 2011). However, this effect is relatively weak as the mice only developed tumors when they reach about 1 year old. Also, this observation could depend on the strain, as another study did not observe this phenotype (Serrano et al., 2013). More evidence links SIRT2 as a tumor activator. SIRT2 deacetylates and stabilizes several oncoproteins. SIRT2 deacetylates and promotes KRAS activity, thereby inducing cell proliferation, colony formation, and tumor growth (Yang et al., 2013). Also, SIRT2 deacetylates K116 of Slug, which subsequently stimulates the growth of basal-like breast cancer (Zhou et al., 2016). In addition, SIRT2-induced c-Myc stabilization promotes pancreatic cancer cell proliferation (Liu et al., 2013). SIRT2 deacetylates and activates LDH-A, which is responsible for lactate production in cancer cell growth (Zhao et al., 2013). In addition, SIRT2 interrupts FOXO1’s interaction with ATG7 and inhibits apoptosis (Zhao et al., 2010). Through removal of long-chain fatty acyl groups on lysine, SIRT2 regulates K-Ras4a transformation activity and promotes ERK via ARF6 (Jing et al., 2017; Kosciuk et al., 2020). With more reports highlighting SIRT2 as a tumor activator, numerous SIRT2 inhibitors have been developed and evaluated in cancer models (Table 3 and Table 4).

The earliest SIRT2 selective inhibitors, AGK2 was developed from high-throughput screening of a small-molecule library (Outeiro et al., 2007). Even though AGK2 treatment showed promising therapeutic effect in cancer studies, its poor aqueous solubility could be problematic to assess its full potency. According to SelleckChem, AGK2 cannot be dissolved in water and ethanol, and only can be dissolved in DMSO with 50°C water bath. Nevertheless, consistent with the SIRT2 knockdown results, AGK2 treatment inhibited the colony formation and induced apoptosis in GB2, GB4, GB11, and GB16 primary glioblastoma cells. In SIRT2 knockdown GB2 cells, AGK2 did not show any antiproliferative effect, which further confirms the SIRT2 selective inhibition of AGK2 in cells (Funato et al., 2018). Due to the toxicity and impermeable blood-brain barrier characteristics of AGK2, another SIRT2 inhibitor, AK7 was tested in GB2 tumor xenograft mice models. After intraperitoneal injection of AK7, a significant impediment of tumor growth was observed. Mice with transplants of GB2 and GB16 SIRT2 knockout cells survived longer and showed less tumorigenicity than mice with transplants GB2 and GB16 SIRT2 wild-type cells. This further confirmed that antitumor effect of AK-7 in this study was through SIRT2 perturbation (Funato et al., 2018). In HCT-116 colorectal cancer cells with wild-type TP53 expression, AGK2 treatment decreased the effects of several chemotherapeutic drugs, including cisplatin, 5-fluorouracil, oxaliplatin, gefitinib, LY294002, and metformin. However, in SW620 colorectal cancer cells with mutant TP53 expression, AGK2 treatment enhanced the anticancer effects of these chemotherapeutic drugs (Yang et al., 2020).

SirReal2 was also shown to effectively decrease the growth of lung, colorectal, lymphoma, gastric, breast, and cervical cancers. SirReal2 decreased the migration and invasion of HGC-27 and MGC-803 gastric cancer cells. SirReal2 inhibited SIRT2 from deacetylating PEPCK1, which promoted degradation of PEPCK1 and decreased mitochondrial metabolism. As a result, the migration of gastric cells was impaired. HGC-27 and MGC-803 with SIRT2 knockdown showed less invasion activities, consistent with the inhibition results. Also, xenograft of SIRT2 knockdown gastric cancer cells in mice formed less metastatic tumors and showed slower growth than that of SIRT2 wild type cells (Li et al., 2018). In a comparison study of SIRT2 inhibitors, SirReal2 showed antiproliferative effects in breast, colorectal, lung, lymphoma, and cervical cancer cells (Spiegelman et al., 2018).

In MCF7 breast cancer cells, the treatment of RK-9123016 increased the acetylated eIF5a level and hindered cell proliferation through degradation of c-Myc (Shah et al., 2016).

Inhibitors with nicotinamide-core impaired proliferation of leukemia and breast cancer cells. NCO-90 and NCO-141 induced apoptosis and mitochondrial superoxide level in leukemic cells, such as HTLV-1-transformed T-cells (Kozako et al., 2018). In S1T, MT-2, Jurkat, and HL60 leukemia cells, NCO-90 and NCO-141 increased acetylation of histone H4, a previously reported SIRT2 substrate, but did not alter acetylation of p53 (Kozako et al., 2018). This confirmed that these compounds inhibited SIRT2, but not SIRT1, in cells. In addition, the treatment of NCO-90 and NCO-141 increased LC-II expression level and autophagosome, which could have induced autophagic cell death (Kozako et al., 2018). KPM-2, a pan SIRT1-3 inhibitor designed from NCO-90, impaired proliferation of MDA-MB-231 breast cancer cells. In the same study, Compound 9, an inhibitor with a similar structure as KPM-2 which shows 11-fold SIRT1 selective inhibition over SIRT2 did not show any antiproliferative effect. Also, Compound 6, a weaker SIRT2 selective inhibitor with a similar structure as KPM-2 showed weaker cytotoxicity than KPM-2. Overall, both results confirmed that the cytotoxicity of KPM-2 is strongly correlated to its SIRT2 inhibition (Mellini et al., 2017).

In PANC-1 pancreatic cancer cells, NPD11033 not only decreased cell proliferation but also increased the acetylation level of eIF5a, a SIRT2 deacetylation substrate (Kudo et al., 2018). Knockdown of SIRT2 in PANC-1 cells also decreased cell proliferation. In addition, an inactive analog RK-0310020 did not show any antiproliferative effect in PANC-1 cells, which further supports that SIRT2 inhibition by NDP11033 induces its anticancer effect (Kudo et al., 2018).

Chroman-4-one and chromone-based Compound 6f and 12a impaired cellular proliferation of MCF7 breast cancer and A549 lung cancer cells. In addition, treatment of Compound 12a in these two cell lines led to cell cycle arrest in G1/G0 phase. Treatment of Compound 6f also showed similar results, but to a smaller extent. In MCF7 cells, both Compound 6f and 12a had increased acetylation level of α-tubulin, a SIRT2 deacetylation target (Seifert et al., 2014).

Compound 35 induced apoptosis in NB4, K562, and MDA-MB-231 cancer cells, and decreased cell proliferation of NB4, Karpas299, and MV4-11 cells (Moniot et al., 2017). Moreover, Compound 39 showed a broader anticancer effect in U937, HL-60, NB4, OCI-AML3, IMS-M2, OCI-AML2, MV4-11, Kasumi-1, and Karpass299 cells (Moniot et al., 2017).

In H441 non-small lung cancer cells, treatment of Compound 24a increased the acetylation level of α-tubulin, and decreased cell proliferation and migration (Yang et al., 2018).

The mechanism-based SIRT2 inhibitors also demonstrated strong anti-cancer effects in cellular and animal models. A mechanism-based SIRT2 inhibitor, TM showed broad anticancer effect in most of the NCI-60 cancer cell lines. These affected cancer cell types include leukemia, non-small lung cancer, colorectal, melanoma, ovarian, renal, prostate, breast, and brain cancer cells. SIRT2 knockdown in MCF7, MDA-MB-468, and MDA-MB-231 breast cancer cells reduced the cell proliferation, confirming that SIRT2 inhibition or perturbation induces cytotoxicity. Interestingly, the control compound, M, which differs from TM just by one atom and could not inhibit SIRT2, does not have anticancer activity. These evidences further confirm that the anticancer activity is through SIRT2 inhibition. The anticancer effect of TM is at least partially through the promotion of c-Myc degradation and SIRT2 knockdown also induced degradation of c-Myc in MCF7 cells. The treatment of TM did not impede the cellular proliferation of MCF-10A and HME1, normal breast epithelial cells. This suggests that TM treatment selectively impacts cancer cell proliferation. The intraperitoneal injection of TM significantly delayed breast tumor growths in MDA-MB-231 xenograft and genetic MMTV-PyMT mouse models without significant weight loss or other obvious toxicity (Jing et al., 2016).

A derivative of TM, AF8 also showed a broad anticancer effect in breast, pancreatic, lung, and colorectal cancer cells. AF8 inhibited the 3D anchorage-independent colony formation of HCT-116 colorectal cancer cells. Furthermore, treatment of AF8 significantly reduced the tumor growth of HCT-116 tumor xenograft mice models in a dose-dependent manner (Farooqi et al., 2019).

A direct comparison of NH4-6, which inhibits SIRT1-3, and NH4-13, which only inhibits SIRT2, showed that selective SIRT2 inhibition could be advantageous when treating cancer. In breast, colorectal, cervical, lung, pancreatic, and glioblastoma cancer cells, low concentrations of NH4-6 and NH4-13 showed weaker cytotoxicity than TM, most likely due to their poor permeability from the charged trimethylammonium moiety. However, in these cancer cells, higher concentrations of both inhibitors showed stronger cytotoxicity than TM, due to their improved aqueous solubility overriding their poor permeability. Furthermore, NH4-6 hindered the cellular proliferation of these cancer cells slightly more potent than NH4-13. In HCT-116 colorectal tumor xenograft mice model, daily treatment of 50 mg/kg NH4-6 caused severe toxicity, while the same dosage of NH4-13 did not alter the overall health. Furthermore, 30 mg/kg every other day injection of NH4-6 and NH4-13 for 2 weeks delayed tumor growth similarly. Daily treatment of 50 mg/kg NH4-13 showed a stronger anticancer effect. Overall, NH4-6 and NH4-13 had similar anticancer effects, but NH4-13, due to its SIRT2 selectivity, has much lower toxicity *in vivo*. Therefore, it could be advantageous to use SIRT2-selective inhibitors to treat cancers (Hong et al., 2021).

Through selective degradation of SIRT2, TM-P4-Thal treatment increased the acetylation level of α-tubulin and fatty acylation level of K-Ras4a. Consequently, TM-P4-Thal showed a stronger antiproliferative effect in MCF7 and MDA-MB-231 breast cancer cells than TM at lower concentrations (Hong et al., 2020).

### SIRT3 Inhibitors in Cancer

SIRT3 regulates various mitochondrial functions, such as ATP generation, metabolism and reactive oxygen species stabilization (Morris, 2013; Hu et al., 2014; Carafa et al., 2016). For example, SIRT3 deacetylates and activates glutamate dehydrogenase, a mitochondrial enzyme that converts glutamate to α-ketoglutarate (Plaitakis et al., 2017; Torrens-Mas et al., 2017). In the beginning, many studies reported SIRT3 as a tumor suppressor. SIRT3 attenuates the stabilization of HIF1α and regulates metabolic reprogramming (Bell et al., 2011; Finley et al., 2011). In breast cancer cell lines, SIRT3 is often less expressed, and the overexpression SIRT3 suppresses glycolysis and cell proliferation (Finley et al., 2011). Patient clinical data also confirmed this trend, as most breast cancer patients had significantly lower SIRT3 expression levels (Alhazzazi et al., 2011). Furthermore, SIRT3 knockout mice developed larger mammary gland tumors than the SIRT3 wild-type mice (Finley et al., 2011).

In contrast, many studies reported SIRT3 as a tumor activator. In bladder cancer cells, SIRT3 deacetylates and inactivates p53, which subsequently promotes cellular proliferation (Li et al., 2010). Also, oral squamous cell carcinoma cells and tissues expressed higher SIRT3 levels (Alhazzazi et al., 2011). Diffusive large B cell lymphomas (DLBCL) required SIRT3 for anaplerotic metabolism, growth, survival, and autophagy. Furthermore, SIRT3 knockdown in DLBCL cells and mice significantly impaired cell proliferation and tumor growth (Li et al., 2019). As such, the role of SIRT3 is likely context and cancer type dependent.

In HNSCC and DLBCL cells, SIRT3 inhibitors have potently hindered cancer growth. By increasing reactive oxygen species (ROS) levels, LC-0296 reduced cell proliferation and promoted apoptosis of UM-SCC-1 and UM-SCC-17B HNSCC cells (Table 5). Meanwhile, LC-0296 did not affect the cell proliferation of normal human oral keratinocytes. Even though these HNSCC cells were resistant to radiation and cisplatin, LC-0296 enhanced the effects of these treatments in HNSCC cells. In UM-SCC-17B cells, LC-2096 increased acetylation levels of NDUFA9 and GDH, SIRT3 deacetylation substrates, and thereby enhanced ROS levels (Alhazzazi et al., 2016).

Treatment of YC8-02 decreased cellular proliferation of OCL-LY1, HBL1, Pfeiffer, SU-DHL4, TMD3, Karpas 422, and OCL-LY7 lymphoma cells (Table 5). In a Karpas 422 tumor xenograft model, YC8-02 significantly impeded the tumor growth. Knockdown of SIRT3 in Karpas422, OCI-LY1, and HBL1 cells impaired cellular proliferation, and xenografted tumors of Karpas422 with knockdown SIRT3 in mice had slower growth. These knockdown results confirmed the therapeutic benefits of YC8-02 and targeting SIRT3 in DLBCLs (Li et al., 2019).

### SIRT5 Inhibitors in Cancer

Many reports showed that SIRT5 has pro-tumor role. The SIRT5 mRNA level is often amplified in tumors compared to normal tissues (Igci et al., 2016; Bringman-Rodenbarger et al., 2018). SIRT5 regulates several metabolic pathways important in cancer, such as glycolysis, TCA, and urea cycle. For instance, SIRT5 demalonylates GAPDH to activate glycolysis (Nishida et al., 2015). Under oxidative stress, SIRT5 desuccinylates PKM2 to decrease the overall carbon flux in TCA cycles (Xiangyun et al., 2017). SIRT5 also activates LDHB, which induces autophagy and cell proliferation of HCT-116 colorectal cancer cells (Shi et al., 2019). Also, overexpression of SIRT5 in hepatocellular carcinoma cells promotes cell proliferation (Zhang et al., 2019). In breast cancer cells, SIRT5 desuccinylates and stabilizes glutaminase, which regulates the overall glutaminolysis, a key metabolic hallmark of cancers (Greene et al., 2019). In MDA-MB-231 and MDA-MB-468 breast, and A-549 lung cancer cells, knockdown of SIRT5 decreases cell proliferation and anchorage-independent growth. Moreover, in mouse xenograft studies, SIRT5-deficient MDA-MB-231 tumors were significantly smaller than wild-type tumors (Greene et al., 2019). In HCT-116 colorectal cancer cells, SIRT5 removes succinyl groups from K393 and K395 of citrate synthase. The hypersuccinylation of citrate synthase decreases cell proliferation and migration, which supports the tumorigenic role of SIRT5 (Ren et al., 2020). Lastly, SIRT5 promotes the proliferation of cutaneous melanoma genotypes, including uveal melanoma. In the A2058 melanoma tumor xenograft model, SIRT5 depletion significantly delayed the tumor growth (Giblin et al., 2021).

There is only one SIRT5 selective inhibitor, DK1-04e, that showed promising effect in cellular and animal cancer studies. In MCF7 and MDA-MB-231 breast cancer cells, treatment of DK1-04e inhibited both cell proliferation and anchorage-independent colony formation (Table 5). Furthermore, treatment with DK1-04e increased mitochondrial global succinylation in MCF7 cells. In both MMTV-PyMT and MDA-MB-231 tumor xenograft mouse models, DK1-04e significantly impaired the tumor growth without any bodyweight loss. The cytotoxicity of DK-104e was dependent on its SIRT5 inhibition. SIRT5 partial knockout in MDA-MB-231 cells have impaired anchorage-independent colony formation. Furthermore, *Sirt5* deletion PyMT mice had slower tumor growth and less metastasis. DK1-04e (O), an inactive derivative with an oxygen atom instead of the sulfur, showed weaker cytotoxicity than DK1-04e. Overall, DK1-04e studies showed that SIRT5 inhibition can be an effective treatment in breast cancer cells (Abril et al., 2021).

### SIRT6 Modulators in Cancer

Through deacetylation and defatty-acylation, SIRT6 regulates numerous biological roles, including cell proliferation, DNA repair, and glucose metabolism (Jing and Lin, 2015; Kosciuk et al., 2019). SIRT6 deacetylates histone H3K9, H3K18, and H3K56 to suppress the activities of several transcriptional factors, such as c-Jun, and NF-κB (Michishita et al., 2008; Kawahara et al., 2009; Michishita et al., 2009; Sundaresan et al., 2012; Tasselli et al., 2016). SIRT6 removes fatty acyl groups from TNF-α to promote its secretion (Jiang et al., 2013). In cancers, SIRT6 is also viewed both as a tumor promoter and a tumor suppressor. As a tumor promoter, SIRT6 promotes cell cycle and tumor proliferation while inhibiting apoptosis (Garcia-Peterson et al., 2017; Huang et al., 2017). In the esophagus, thyroid, and melanocytes, SIRT6 is expressed higher than in normal tissues (Huang et al., 2017). As a tumor suppressor, SIRT6 is down-regulated in colorectal, ovarian, breast, lung, pancreatic, and hepatocellular tumors (Marquardt et al., 2013; Zhang et al., 2015; Kugel et al., 2016). SIRT6 attenuates migration and invasion of ovarian cancer cells (Bae et al., 2018). SIRT6 deficient MEF cells proliferate faster than control wild-type cells and loss of SIRT6 induced faster tumor formation in mice (Sebastian et al., 2012). In colorectal cancer stem cells, SIRT6 impaired cellular proliferation and anchorage-independent colony formation (Sebastian et al., 2012). Furthermore, through defatty-acylating, SIRT6 regulates R-Ras2 localization, and subsequently hinders cell proliferation (Zhang et al., 2017).

The SIRT6 modulators’ effects in cancer cells are summarized in Table 6. It is only recently that UBCS039 and MDL-800 were reported to activate SIRT6 and decrease proliferation of lung, hepatocellular carcinoma, and pancreatic cancer cells. For instance, UBCS039 induced autophagosome accumulation, thereby leading to apoptosis. UBCS060, an inactive analog of UBCS039, could not increase the autophagosome accumulation and autophagy-induced apoptosis (You et al., 2017). A previous report suggested that lack of SIRT6 decreased oxygen consumption and ATP level in the heart (Khan et al., 2018). In accordance with this, treatment of UBSC039 activated ROS production and increased ATP level in H1299 and HeLa cells (Iachettini et al., 2018).

MDL-800 significantly suppressed proliferation of BEL7405 cells *in vitro* and in mouse xenograft studies (Huang et al., 2018). MDL-800 promoted cell cycle arrest in G0/G1 phase, as p21 and p27 expressions have increased, and CDK2, CDK4, cyclin D1, and cyclin D3 levels have decreased. To confirm whether the effect of MDL-800 depended on SIRT6 activation, SIRT6 knockout BEL6405 cells were treated with MDL-800. In these SIRT6 knockout cells, treatment of MDL-800 did not change any of the previously observed markers for the cell cycle arrest, confirming that the effect of MDL-800 was through SIRT6 activation (Huang et al., 2018). In addition to the hepatocellular carcinoma cells, MDL-800 inhibited the proliferation of 12 non-small cell lung cancer (NSCLC) cells from the NCI-60 screening. MDL-800 did not affect the proliferation of SIRT6-knockout HCC827 and PC9 NSCLC cells, which confirmed the on-target activation of SIRT6 by MDL-800. In the HCC827 tumor xenograft mouse study, administration of MDL-800 increased histone H3 acetylation and significantly decreased the tumor growth (Shang et al., 2021).

In specific conditions, several SIRT6 inhibitors showed antiproliferative effects. As mentioned, Compound 2, 3, and 8 increased H3K9 acetylation in BxPC-3 pancreatic cancer cells (Sociali et al., 2015). Also, Compound 3 and 8 increased the glucose uptake in both BxPC-3 and L6 myoblasts. Among these three, only Compound 8 showed antiproliferative effect against BxPC-3 cells. Interestingly, Compound 2 and 3 showed synergistic effect with gemcitabine against proliferation of BxPC-3 cells (Sociali et al., 2015).

Compound 5 and 11 promoted glucose uptake and inhibited TNF-α production. Even though Compound 5 and 11 were not toxic, both SIRT6 inhibitors with gemcitabine showed a stronger anticancer effect in BxPC-3 cell proliferation. In the pharmacokinetics study, as Compound 5 showed a relatively short half-life, additional modifications on this compound are needed to improve the bioavailability, which will allow more accurate assessment in animal studies (Damonte et al., 2017).

### Pan-Sirtuin Inhibitors in Cancer

In addition to the selective sirtuin inhibitors, numerous pan-sirtuin inhibitors were reported to decrease cancer cell proliferation. However, because treatment of these pan-sirtuin inhibitors may cause over-toxicity issues, extra caution is needed when using these inhibitors. In NCI-H460 lung cancer and HeLa cervical cancer cells, Cambinol increased acetylation levels of several sirtuin substrates, including p53, α-tubulin, FOXO3a, and Ku70 (Heltweg et al., 2006). In RPMI8226 and U266 multiple myeloma cells, Cambinol induced apoptosis, cell proliferation impairment, and cell cycle arrest by increasing p53, p21, cleaved PARP, and cleaved caspase 3 (Lu et al., 2021). In orthopedic tumor xenograft mice model with HepG2 hepatocarcinoma cells, Cambinol significantly reduced tumor growth, which was consistent with the SIRT1 knockdown results of *in vivo* intrahepatic xenograft mouse model (Portmann et al., 2013). Also, it was reported that SIRT1 stabilizes N-Myc protein and promote neuroblastoma cell proliferation. Thus, the knockout SIRT1 BE (2)-C cells had lower N-Myc level than the wild-type cells. In accordance with this, Cambinol treatment in TH-MYCN transgenic mice had decreased neuroblastoma formation (Marshall et al., 2011; Portmann et al., 2013). In HepG2 and Huh7 hepatocarcinoma cells, compared to a single treatment of sorafenib, a combination of Cambinol and sorafenib showed an enhanced effect in reducing cell proliferation, migration, and invasion (Ceballos et al., 2021).

In MCF7 breast and H1299 non-small lung cancer cells, treatment with Sirtinol led to senescence-like growth arrest and decreased activation of the RAS-MAPK pathway. Similar results were also observed with SIRT1 knockdown (Ota et al., 2006). Furthermore, Sirtinol reduced cell proliferation of H1299 non-small lung, PC3 prostrate, DU145 prostate, HeLa cervical, S1T adult T-cell leukemia/lymphoma (ATL), and Jurkat ATL cancer cells (Kojima et al., 2008; Kozako et al., 2012; Fong et al., 2014). In PC3, DU145, S1T and Jurkat cells, knockdown of SIRT1 also hindered cell proliferation (Kojima et al., 2008; Kozako et al., 2012). Combination treatment of sodium dichloroacetic acid (DCA) and Sirtinol led to synergistic anticancer effect in A549 and H129 NSCLC cells *in vitro*, and *in vivo* A549 tumor xenograft mice model (Ma et al., 2018).

Through SIRT1 inhibition, Salermide induced apoptosis in MOL4 acute lymphoblastic leukemia, SW480 colorectal, KG-1a acute myelogenous leukemia, and Raji Burkitt’s lymphoma cells. Based on the knockdown studies of SIRT1 and SIRT2, Salermide-induced apoptosis is mainly through its SIRT1 inhibition (Lara et al., 2009). Furthermore, Salermide showed strong anti-proliferative effects in BE (2)-C neuroblastoma and MIA-PaCa-2 pancreatic cancer cells, consistent with the results from SIRT2 knockdown. Furthermore, SIRT2 knockdown and 50 μM of Salermide in these cell lines induced n-Myc and c-Myc degradation (Liu et al., 2013).

Even though Tenovin-6 showed strong anti-cancer potency, Tenovin-6 usage may be limited due to its off-target effect and potential over-toxicity issues, as mentioned earlier. In both cellular and tumor xenograft mice studies, Tenovin-6 showed strong antiproliferative effects against ARN8 melanoma cells (Lain et al., 2008). In AGS, AGS-EBV, SNU-179, HGC-27, N87, SNU-1, and KATO-III gastric cancer cells, Tenovin-6 decreased the cell proliferation. Moreover, Tenovin-6 hindered cell proliferation and anchorage-independent growth of AGS, AGS-EBV, and HGC-27 through increasing acetyl p53 levels (Ke et al., 2020). In A549 NSLCL cells, a combination of Tenovin-6 and metformin demonstrated a synergistic antiproliferative effect by HIC1-dependent SIRT1 level reduction (Lee et al., 2019). By increasing the expression of p53 and ROS level, Tenovin-6 also attenuated migration and proliferation of 92.1, Mel-270, Omm-1, and Omm-2.3 uveal melanoma (UM) cells. Also, in 92.1, and Mel-270 cells, Tenovin-6 had a synergistic effect with Vinblastine, a chemotherapeutic agent for UM patients (Dai et al., 2016).

BZD9L1 showed antiproliferative effects in HCT-116 colorectal, CCRF-CEM leukemia, and MDA-MB-468 breast cancer cells (Tan et al., 2018). Furthermore, in HCT-116 and HT-29 colorectal cancer cells, BZD9L1 significantly decreased the cell migration and anchorage-independent growth (Tan et al., 2018). Only in HCT-116 cells, BZD9L1 showed a synergistic anticancer effect with 5-Fluorouracil, a conventional chemotherapeutic agent. The combination of BZD9L1 and 5-Fluorouracil increased cell cycle, arrest, and apoptosis, while it decreased the spheroid proliferation. In addition, the combination treatment of BZD9L1 and 5-Fluorouracil significantly impaired tumor growth of HCT-116 in a tumor xenograft mouse study (Tan et al., 2019).

In U373 and Hs683 glioma cells, treatment of Compound 18 increased acetylation levels of histone H4, histone H3K56, and α-tubulin, which confirmed cellular inhibition of SIRT1 and SIRT2 (Schnekenburger et al., 2017). Consistent with the SIRT1 and SIRT2 knockdown results, Compound 18 impaired cell proliferation of U373 and Hs683 cells. Moreover, Compound 18 showed a broad anticancer effect, as its average GI_50_ was about 3 μM in the NCI-60 screening. In the zebrafish xenotransplant model, Compound 18 treatment significantly reduced the growth of fluorescent-labeled HS683 and U373 tumors (Schnekenburger et al., 2017).

Compound 3g exerted stronger cytotoxicity than EX-527 in several cancer cell lines, K562 leukemia, HCT-116 colorectal, HT-29 colorectal, H460 lung, A549 lung, and MCF7 breast cancer cells. Such increased potency of Compound 3g from EX-527 could come from the dual inhibition of SIRT1 and SIRT2, but this was not confirmed in the study. Thus, future studies proving cellular inhibition of SIRT1 and SIRT2 by Compound 3g will be needed (Laaroussi et al., 2020).

A SIRT1-3 inhibitor, MC2494 decreased metabolic activity and proliferation of U937 leukemia cells (Carafa et al., 2020). MC2494 decreased ATP production and expression levels of PGC1α and PGC1β, which are important for metabolic regulation. In addition, PGC1α is present in the cytoplasm under normal condition, but more perinuclear PGC1α was detected, after treatment of MC2494 (Carafa et al., 2020).

JH-T4, a mechanism-based SIRT1-3 inhibitor, portrayed strong antiproliferative effects in a wide range of cancer cells, including MCF7 breast, MDA-MB-231 breast, HCT-116 colorectal, and NCI-H23 lung cancer cells. However, JH-T4 also affected the proliferation of normal epithelial cells like HME1 and MCF-10A. Thus, usage of JH-T4 may cause an over-toxicity problem in animal studies (Spiegelman et al., 2019).

## Sirtuin Modulators in Neurological Diseases

### SIRT1 Inhibitors in Neurological Diseases

Both protective and detrimental effects of SIRT1 in neurological diseases have been reported. SIRT1 inhibits neurogenesis through inhibiting the transcriptional factors Hes1 and Mash1 (Prozorovski et al., 2008). SIRT1 maintains cognitive level and synaptic plasticity (Michan et al., 2010). The brain of SIRT1 knockout mice looked normal but showed a significant decrease in dendritic extension, length, and complexity (Michan et al., 2010). SIRT1 is also reported to promote neurite outgrowth through suppressing expression and phosphorylation of mTOR (Guo et al., 2011). The parietal cortex of Alzheimer’s disease patients showed lower expression of SIRT1, which may be connected to an increase of β-amyloid and tau (Julien et al., 2009). In microglial cells, overexpression of SIRT1 decreased acetylation of RelA/p65 subunit of NF-κβ, which consequently inhibited NF-κβ signaling induced by Amyloid-β and neuronal death (Chen et al., 2005). Overexpression of SIRT1 decreases acetylation of FOXO3a, and consequently protects against huntingtin toxicity (Jeong et al., 2011). These reports highlights the beneficial effects of SIRT1 in the neurological system.

In contrast, other reports also point to the negative impacts of SIRT1 in neurological diseases. Knockdown of SIRT1 fostered neurogenesis of P19 embryonic carcinoma cells. SIRT1 inhibition by EX-527 also promoted the differentiation of P19 cells into functional neurons with around 50% efficiency (Kim et al., 2016). In single prolonged stress (SPS) mice mimicking post-traumatic stress disorder (PTSD), *Sirt1* deleted mice had less anxiety and freezing time, which indicated SIRT1 as a potential therapeutic target for PTSD. Osmotic delivery of EX-527 to ventral CA1 of hippocampus had deactivated helix-loop-helix transcription factor 2 and subsequently hindered the expression of MAO-A. This further led to the stabilization of serotonin. In addition, EX-527 ensured normal neuronal plasticity by decreasing dendritic spines and abnormal shapes (Li et al., 2019). Injection of EX-527 to the ventrolateral orbital cortex had ameliorated morphine addiction of rats. Morphine injected rats had elevated SIRT1 expression level, which got diminished with the administration of EX-527 (Wei et al., 2021). Lastly, in the rat model of middle cerebral artery occlusion, which simulates cerebral ischemia-reperfusion injury, EX-527 enhanced the survival rate and decreased cerebral infarction volume (Nikseresht et al., 2019). Lastly, EX-527 was tested in a clinical trial with patients with Huntington’s Disease. EX-527 did not cause any adverse side-effect, but in a short 12 week trial, EX-527 did not affect the huntingtin level (Sussmuth et al., 2015). No further clinical study with EX-527 have been reported.

In the subarachnoid hemorrhage rat model, Sirtinol treatment lowered SIRT1 expression, which further induced damage of the blood-brain barrier and neurological ability. In addition, Sirtinol aggravated brain edema and increased endothelial cell apoptosis (Zhou et al., 2014). Thus, for subarachnoid hemorrhage, a validated potent SIRT1 activator should be tested as a potential treatment.

### SIRT2 Inhibitors in Neurological Diseases

Through deacetylation of α-tubulin and activation of the CREB signaling pathway, SIRT2 promotes neuronal differentiation of mesenchymal stem cells (Jeong and Cho, 2017). In oligodendroglia and myelin sheets, SIRT2 is often highly expressed (Li et al., 2007). Through increasing the expression level of myelin basic proteins, SIRT2 boosts oligodendroglia differentiation (Ji et al., 2011). Also, because of the increased acetylated FOXO3a level and decreased Bim expression, SIRT2 knockout mice showed resistance against 1-methyl-4-phenyl-1,2,3,6-tetrahydropyridine (MPTP), which induces neurotoxicity like Parkinson’s Disease (Liu et al., 2014).

In accordance with the role of SIRT2, many SIRT2 inhibitors have been reported to ameliorate the symptoms from neurological disease. The SIRT2 inhibitor AGK2 showed a neuroprotective effect in Parkinson’s Disease models. Releasing adenylate kinase to the media, AGK2 reduced α-Synuclein-mediated toxicity. α-Synuclein toxicity was decreased by SIRT2 knockdown in neuroglioma cells, which verified that the effect of AGK2 was from its SIRT2 inhibition. Also, AGK2 treatment increased viabilities of dopamine neurons in cellular and drosophila models (Outeiro et al., 2007). In mice, AGK2 ameliorated lipopolysaccharides (LPS)-induced neuroinflammation, decreasing LPS-induced CD11b TNF-α, and IL-6 levels. AGK2 treatment in mice decreased TUNEL signals, which are indicators of brain apoptotic damage (Wang et al., 2016). In the middle cerebral artery occlusion (MCAO) mice model simulating focal ischemic stroke, AGK2 administration lowered cleaved-caspase 3, Bim, and Bad, which consequently hindered apoptosis (She et al., 2018). Lastly, in cultured hippocampal neurons, AGK2 protected cell deaths from exposure to H_2_O_2_. Also, in the same study, AGK2 promoted VEGF and HO-1 mRNA levels, which stimulates neuroprotection against ischemic injury. This result was consistent with that from the experiments with SIRT2 knockout DT40 cells (Kaitsuka et al., 2020).

In the MCAO mouse model, the SIRT2-selective inhibitor AK-7 decreased the infarction volume and promoted neurological recovery. Moreover, AK-7 increased the activation of a MAP kinase, p38, *in vitro* and *in vivo*, which led to the neuroprotection from the ischemic injury. Knockdown of SIRT2 also activated p38 in Neuro-2a cells (Wu et al., 2018). In the microglia from the sevoflurane-treated neonatal rat study, AK-7 decreased pro-inflammatory markers, while increasing anti-inflammatory markers. Sevoflurane is used as an inhalational anesthetic, which could damage the developing brain (Wu et al., 2020).

A nicotinamide-derived SIRT2 selective inhibitor, NCO-141 treatment had increased spatial learning and memory deficiency of 5 month-old senesce-accelerated mouse prone-8 (SAMP8) mice, which mimics Alzheimer’s disease. In SAMP8 mice, treatment with a selective SIRT2 inhibitor NCO-141 did not indicate any therapeutic benefits. Nevertheless, NCO-141 increased glutamate receptor subunits GluN2A, GluN2B, and GluA1, which are essential for synaptic plasticity. To confirm whether NCO-141 inhibited SIRT2 in hippocampus, ATP-binding cassette transporter Abca1 expression level was measured, as transcription of Abca1 is inhibited by SIRT2. As expected, NCO-141 treated SAMP8 mice elevated level of Abca1 (Diaz-Perdigon et al., 2020). NCO-90-based SIRT2 inhibitor Compound 53 and pan SIRT1-3 inhibitor KPM-2 significantly promoted neurite outgrowth of Neuro-2a cells (Mellini et al., 2017; Mellini et al., 2019).

### SIRT6 Inhibitors in Neurological Diseases

SIRT6 regulates stem cell differentiation and neuroectoderm development through its deacetylation of histone H3. SIRT6 knockout mice showed higher expressions of Oct4, Sox2, and Nanog, which are important for stem cell pluripotency. Consequently, this led to higher expression of Tet enzymes, which produces 5-hydroxymethylcytosine. With increased 5-hydroxymethylcytosine, more embryonic stem cells differentiated into neuroectoderm. When SIRT6 was present, expressions of Oct4, Sox2, and Nanog were repressed, and led to balanced differentiation of embryonic stem cells (Etchegaray et al., 2015). For immunity and inflammation, SIRT6 de-fatty acylates TNFα and promotes its secretion. This could potentially regulate inflammatory cytokine production and necrosis (Jiang et al., 2013). Mice with brain-specific SIRT6 knockout showed behavioral abnormalities along with DNA damage and increased phosphorylated Tau. Also, in patients with Alzheimer’s disease, lower expression of SIRT6 was measured, which hints the neuroprotective role of SIRT6 (Kaluski et al., 2017). Lastly, SIRT6 promotes the differentiation of dendritic cells *in vitro* and *in vivo* (Lasiglie et al., 2016).

Compound 1 is a SIRT6 inhibitor that had shown therapeutic effect in autoimmune encephalomyelitis (EAE), an animal model of multiple sclerosis (Ferrara et al., 2020). In a previous study, Compound 1 increased glucose uptake and GLUT-1 expression, and decreased TNF-α in BxPC-3 pancreatic cancer cells. These observations indicate a potent cellular SIRT6 inhibition by Compound 1 (Parenti et al., 2014). In C57bl/6 mice with MOG35-55 injection, which mimics EAE conditions, treatment with Compound 1 decreased the levels of TNFα and neurological impairments (Ferrara et al., 2020).

Since SIRT6 inhibitors may affect the development of neurological disorder, assessing SIRT6 activators in neurological disease models will be interesting, but so far there has been no report on this direction.

## Sirtuin Modulators in Cardiovascular Diseases

In addition to cancer and neurological disease, several sirtuins have been connected to cardiovascular diseases, like vascular aging, atherosclerosis, cardiac hypertrophy, and many more (Alcendor et al., 2004; Alcendor et al., 2007; Zhang et al., 2008; Balestrieri et al., 2015; D'Onofrio et al., 2016; Liu et al., 2016). Among these, several sirtuin modulators were specifically evaluated in cardiovascular diseases related to cardiomyocytes. Thus, we have summarized these SIRT1/2 and SIRT6 inhibitors.

### SIRT1/2 Inhibitors in Cardiovascular Diseases

Mice lacking SIRT1 possess congenital cardiac abnormalities, and most could not survive beyond two weeks (Cheng et al., 2003). Also, SIRT1 deacetylates and regulates sodium channel Nav1.5. Deficiency of SIRT1 decreased expression of Nav1.5 in the cardiomyocyte membrane and induced cardiac abnormalities (Vikram et al., 2017). Low to moderate overexpression of SIRT1 in transgenic mouse inhibited fibrosis and cardiac hypertrophy. However, high overexpression of SIRT1 aggravated hypertrophy (Alcendor et al., 2007).

After Sirtinol treatment, neonatal rats showed a decrease in cardiomyocytes. In the same model, SIRT1 overexpression increased cardiomyocytes. Also, in the late phase of cardiac ischemia preconditioning in rats, treatment of Sirtinol significantly increased the infarct size, which made them more prone to the ischemia injury (Safari et al., 2017). Splitomicin, a yeast sirtuin inhibitor, had promoted carotid artery thrombus formation in a photochemical injury mouse study. In human endothelial cells, both SIRT1 siRNA and Splitomicin had increased and activated tissue factor protein, which promotes coagulation and thrombus formation. As the SIRT1 inhibitor worsens cardiovascular diseases, a reliable SIRT1 activator may be needed for the therapeutic benefit (Breitenstein et al., 2011). The potential cardiovascular effect of SIRT1 inhibitors may also limit the use of them for treating other diseases.

### SIRT6 Inhibitors in Cardiovascular Diseases

SIRT6 suppresses IGF-Akt signaling, which promotes heart failure when activated. SIRT6 knockout mice promoted cardiac hypertrophy upon hypertrophic stimulus (Sundaresan et al., 2012). In a mouse model of transverse aortic constriction (TAC)-induced heart failure, SIRT6 maintained telomere integrity, thereby decreasing cardiac fibrosis and infarct size (Li et al., 2017).

Consistent with the role of SIRT6 to prevent cardiovascular diseases, a SIRT6 inhibitor, OSS-128267, intensified diabetic cardiomyopathy (DCM). In a separate study using BxPC-3 pancreatic cells, OSS-128267 increased glucose uptake and GLUT-1 expression, and decreased TNF-α, which suggests potent inhibition of SIRT6 (Parenti et al., 2014). In the mouse model of streptozotocin-induced diabetes and high glucose-treated cardiomyocytes, OSS-129167 promoted inflammation and oxidative stress, which led to diabetes-induced cardiomyocyte apoptosis (Huang et al., 2021). In this DCM disease model, treatment of SIRT6 activators like MDL-800 or UBCS039 may be beneficial.

## Concluding Remarks

In this review, we have summarized the effects of various sirtuin modulators in cancer, neurological, and cardiovascular diseases. We anticipate that this review can help the readers to choose a suitable sirtuin modulator in different disease models. Overall, although there may be a few contradicting reports, some general trends can be extracted from the majority of the literature. SIRT2 and SIRT5 inhibitors showed rather consistent and promising effect in treating cancers. SIRT2 inhibitors have also showed beneficial effects in neurological diseases. On the other hand, SIRT1 and SIRT6 inhibitors have aggravated cardiovascular diseases, which underlines the need for a reliable SIRT1 and SIRT6 activators. These generalized trends support that the development of sirtuin modulators with enhanced potency and selectivity will be essential to further validate the preclinical data and explore the potential for treating various human diseases.

## References

[B1] Abril Y. L. N. Fernandez I. R. Hong J. Y. Chiang Y. L. Kutateladze D. A. Zhao Q. (2021). Pharmacological and Genetic Perturbation Establish SIRT5 as a Promising Target in Breast Cancer. Oncogene 40, 1644–1658. 10.1038/s41388-020-01637-w 33479498 PMC7935767

[B2] Aksoy P. White T. A. Thompson M. Chini E. N. (2006). Regulation of Intracellular Levels of NAD: a Novel Role for CD38. Biochem. Biophys. Res. Commun. 345, 1386–1392. 10.1016/j.bbrc.2006.05.042 16730329

[B4] Alcendor R. R. Gao S. Zhai P. Zablocki D. Holle E. Yu X. (2007). Sirt1 Regulates Aging and Resistance to Oxidative Stress in the Heart. Circ. Res. 100, 1512–1521. 10.1161/01.RES.0000267723.65696.4a 17446436

[B3] Alcendor R. R. Kirshenbaum L. A. Imai S. Vatner S. F. Sadoshima J. (2004). Silent Information Regulator 2alpha, a Longevity Factor and Class III Histone Deacetylase, Is an Essential Endogenous Apoptosis Inhibitor in Cardiac Myocytes. Circ. Res. 95, 971–980. 10.1161/01.RES.0000147557.75257.ff 15486319

[B6] Alhazzazi T. Y. Kamarajan P. Joo N. Huang J. Y. Verdin E. D'Silva N. J. (2011). Sirtuin-3 (SIRT3), a Novel Potential Therapeutic Target for Oral Cancer. Cancer 117, 1670–1678. 10.1002/cncr.25676 21472714 PMC3117020

[B5] Alhazzazi T. Y. Kamarajan P. Verdin E. Kapila Y. L. (2011). SIRT3 and Cancer: Tumor Promoter or Suppressor? Biochim. Biophys. Acta 1816, 80–88. 10.1016/j.bbcan.2011.04.004 21586315 PMC3129516

[B7] Alhazzazi T. Y. Kamarajan P. Xu Y. Ai T. Chen L. Verdin E. (2016). A Novel Sirtuin-3 Inhibitor, LC-0296, Inhibits Cell Survival and Proliferation, and Promotes Apoptosis of Head and Neck Cancer Cells. Anticancer Res. 36, 49–60. 26722027 PMC5417072

[B8] Anderson K. A. Huynh F. K. Fisher-Wellman K. Stuart J. D. Peterson B. S. Douros J. D. (2017). SIRT4 Is a Lysine Deacylase that Controls Leucine Metabolism and Insulin Secretion. Cell Metab. 25, 838–e15. 10.1016/j.cmet.2017.03.003 28380376 PMC5444661

[B9] Asaka R. Miyamoto T. Yamada Y. Ando H. Mvunta D. H. Kobara H. (2015). Sirtuin 1 Promotes the Growth and Cisplatin Resistance of Endometrial Carcinoma Cells: a Novel Therapeutic Target. Lab. Invest. 95, 1363–1373. 10.1038/labinvest.2015.119 26367491

[B10] Avalos J. L. Bever K. M. Wolberger C. (2005). Mechanism of Sirtuin Inhibition by Nicotinamide: Altering the NAD(+) Cosubstrate Specificity of a Sir2 Enzyme. Mol. Cell 17, 855–868. 10.1016/j.molcel.2005.02.022 15780941

[B11] Bae J. S. Noh S. J. Kim K. M. Park S. H. Hussein U. K. Park H. S. (2018). SIRT6 Is Involved in the Progression of Ovarian Carcinomas via β-Catenin-Mediated Epithelial to Mesenchymal Transition. Front. Oncol. 8, 538. 10.3389/fonc.2018.00538 30524965 PMC6256124

[B12] Balestrieri M. L. Rizzo M. R. Barbieri M. Paolisso P. D'Onofrio N. Giovane A. (2015). Sirtuin 6 Expression and Inflammatory Activity in Diabetic Atherosclerotic Plaques: Effects of Incretin Treatment. Diabetes 64, 1395–1406. 10.2337/db14-1149 25325735

[B13] Beher D. Wu J. Cumine S. Kim K. W. Lu S. C. Atangan L. (2009). Resveratrol Is Not a Direct Activator of SIRT1 Enzyme Activity. Chem. Biol. Drug Des. 74, 619–624. 10.1111/j.1747-0285.2009.00901.x 19843076

[B14] Bell E. L. Emerling B. M. Ricoult S. J. Guarente L. (2011). SirT3 Suppresses Hypoxia Inducible Factor 1α and Tumor Growth by Inhibiting Mitochondrial ROS Production. Oncogene 30, 2986–2996. 10.1038/onc.2011.37 21358671 PMC3134877

[B15] Bheda P. Jing H. Wolberger C. Lin H. (2016). The Substrate Specificity of Sirtuins. Annu. Rev. Biochem. 85, 405–429. 10.1146/annurev-biochem-060815-014537 27088879

[B16] Blank M. F. Chen S. Poetz F. Schnölzer M. Voit R. Grummt I. (2017). SIRT7-dependent Deacetylation of CDK9 Activates RNA Polymerase II Transcription. Nucleic Acids Res. 45, 2675–2686. 10.1093/nar/gkx053 28426094 PMC5389538

[B17] Bonkowski M. S. Sinclair D. A. (2016). Slowing Ageing by Design: the Rise of NAD+ and Sirtuin-Activating Compounds. Nat. Rev. Mol. Cell Biol. 17, 679–690. 10.1038/nrm.2016.93 27552971 PMC5107309

[B18] Borra M. T. Smith B. C. Denu J. M. (2005). Mechanism of Human SIRT1 Activation by Resveratrol. J. Biol. Chem. 280, 17187–17195. 10.1074/jbc.M501250200 15749705

[B19] Borradaile N. M. Pickering J. G. (2009). NAD(+), Sirtuins, and Cardiovascular Disease. Curr. Pharm. Des. 15, 110–117. 10.2174/138161209787185742 19149606

[B20] Bosch-Presegué L. Vaquero A. (2011). The Dual Role of Sirtuins in Cancer. Genes Cancer 2, 648–662. 10.1177/1947601911417862 21941620 PMC3174263

[B21] Breitenstein A. Stein S. Holy E. W. Camici G. G. Lohmann C. Akhmedov A. (2011). Sirt1 Inhibition Promotes *In Vivo* Arterial Thrombosis and Tissue Factor Expression in Stimulated Cells. Cardiovasc. Res. 89, 464–472. 10.1093/cvr/cvq339 20978007

[B22] Bringman-Rodenbarger L. R. Guo A. H. Lyssiotis C. A. Lombard D. B. (2018). Emerging Roles for SIRT5 in Metabolism and Cancer. Antioxid. Redox Signal 28, 677–690. 10.1089/ars.2017.7264 28707979 PMC5824490

[B24] Carafa V. Nebbioso A. Cuomo F. Rotili D. Cobellis G. Bontempo P. (2018). RIP1-HAT1-SIRT Complex Identification and Targeting in Treatment and Prevention of Cancer. Clin. Cancer Res. 24, 2886–2900. 10.1158/1078-0432.CCR-17-3081 29535128

[B23] Carafa V. Rotili D. Forgione M. Cuomo F. Serretiello E. Hailu G. S. (2016). Sirtuin Functions and Modulation: from Chemistry to the Clinic. Clin. Epigenetics 8, 61. 10.1186/s13148-016-0224-3 27226812 PMC4879741

[B25] Carafa V. Russo R. Della Torre L. Cuomo F. Dell'Aversana C. Sarno F. (2020). The Pan-Sirtuin Inhibitor MC2494 Regulates Mitochondrial Function in a Leukemia Cell Line. Front. Oncol. 10, 820. 10.3389/fonc.2020.00820 32528892 PMC7255067

[B26] Ceballos M. P. Angel A. Delprato C. B. Livore V. I. Ferretti A. C. Lucci A. (2021). Sirtuin 1 and 2 Inhibitors Enhance the Inhibitory Effect of Sorafenib in Hepatocellular Carcinoma Cells. Eur. J. Pharmacol. 892, 173736. 10.1016/j.ejphar.2020.173736 33220273

[B27] Cha Y. Han M. J. Cha H. J. Zoldan J. Burkart A. Jung J. H. (2017). Metabolic Control of Primed Human Pluripotent Stem Cell Fate and Function by the miR-200c-SIRT2 axis. Nat. Cell Biol. 19, 445–456. 10.1038/ncb3517 28436968 PMC5545746

[B28] Chalkiadaki A. Guarente L. (2015). The Multifaceted Functions of Sirtuins in Cancer. Nat. Rev. Cancer 15, 608–624. 10.1038/nrc3985 26383140

[B31] Chen G. Zhang B. Xu H. Sun Y. Shi Y. Luo Y. (2017). Suppression of Sirt1 Sensitizes Lung Cancer Cells to WEE1 Inhibitor MK-1775-Induced DNA Damage and Apoptosis. Oncogene 36, 6863–6872. 10.1038/onc.2017.297 28869605

[B32] Chen J. Cao L. Li Z. Li Y. (2019). SIRT1 Promotes GLUT1 Expression and Bladder Cancer Progression via Regulation of Glucose Uptake. Hum. Cell 32, 193–201. 10.1007/s13577-019-00237-5 30868406

[B30] Chen J. Zhou Y. Mueller-Steiner S. Chen L. F. Kwon H. Yi S. (2005). SIRT1 Protects against Microglia-dependent Amyloid-Beta Toxicity through Inhibiting NF-kappaB Signaling. J. Biol. Chem. 280, 40364–40374. 10.1074/jbc.M509329200 16183991

[B29] Chen L. F. Mu Y. Greene W. C. (2002). Acetylation of RelA at Discrete Sites Regulates Distinct Nuclear Functions of NF-kappaB. EMBO J. 21, 6539–6548. 10.1093/emboj/cdf660 12456660 PMC136963

[B33] Cheng H. L. Mostoslavsky R. Saito S. Manis J. P. Gu Y. Patel P. (2003). Developmental Defects and P53 Hyperacetylation in Sir2 Homolog (SIRT1)-Deficient Mice. Proc. Natl. Acad. Sci. U. S. A. 100, 10794–10799. 10.1073/pnas.1934713100 12960381 PMC196882

[B39] D'Onofrio N. Servillo L. Giovane A. Casale R. Vitiello M. Marfella R. (2016). Ergothioneine Oxidation in the protection against High-Glucose Induced Endothelial Senescence: Involvement of SIRT1 and SIRT6. Free Radic. Biol. Med. 96, 211–222. 10.1016/j.freeradbiomed.2016.04.013 27101740

[B34] Dai H. Kustigian L. Carney D. Case A. Considine T. Hubbard B. P. (2010). SIRT1 Activation by Small Molecules: Kinetic and Biophysical Evidence for Direct Interaction of Enzyme and Activator. J. Biol. Chem. 285, 32695–32703. 10.1074/jbc.M110.133892 20702418 PMC2963390

[B36] Dai H. Sinclair D. A. Ellis J. L. Steegborn C. (2018). Sirtuin Activators and Inhibitors: Promises, Achievements, and Challenges. Pharmacol. Ther. 188, 140–154. 10.1016/j.pharmthera.2018.03.004 29577959 PMC6342514

[B35] Dai W. Zhou J. Jin B. Pan J. (2016). Class III-specific HDAC Inhibitor Tenovin-6 Induces Apoptosis, Suppresses Migration and Eliminates Cancer Stem Cells in Uveal Melanoma. Sci. Rep. 6, 22622. 10.1038/srep22622 26940009 PMC4778058

[B37] Damonte P. Sociali G. Parenti M. D. Soncini D. Bauer I. Boero S. (2017). SIRT6 Inhibitors with Salicylate-like Structure Show Immunosuppressive and Chemosensitizing Effects. Bioorg. Med. Chem. 25, 5849–5858. 10.1016/j.bmc.2017.09.023 28958848

[B38] Diaz-Perdigon T. Belloch F. B. Ricobaraza A. Elboray E. E. Suzuki T. Tordera R. M. (2020). Early Sirtuin 2 Inhibition Prevents Age-Related Cognitive Decline in a Senescence-Accelerated Mouse Model. Neuropsychopharmacology 45, 347–357. 10.1038/s41386-019-0503-8 31471557 PMC6901465

[B40] Du J. Zhou Y. Su X. Yu J. J. Khan S. Jiang H. (2011). Sirt5 Is a NAD-dependent Protein Lysine Demalonylase and Desuccinylase. Science 334, 806–809. 10.1126/science.1207861 22076378 PMC3217313

[B41] Escande C. Nin V. Price N. L. Capellini V. Gomes A. P. Barbosa M. T. (2013). Flavonoid Apigenin Is an Inhibitor of the NAD+ Ase CD38: Implications for Cellular NAD+ Metabolism, Protein Acetylation, and Treatment of Metabolic Syndrome. Diabetes 62, 1084–1093. 10.2337/db12-1139 23172919 PMC3609577

[B42] Etchegaray J. P. Chavez L. Huang Y. Ross K. N. Choi J. Martinez-Pastor B. (2015). The Histone Deacetylase SIRT6 Controls Embryonic Stem Cell Fate via TET-Mediated Production of 5-hydroxymethylcytosine. Nat. Cell Biol. 17, 545–557. 10.1038/ncb3147 25915124 PMC4593707

[B43] Farooqi A. S. Hong J. Y. Cao J. Lu X. Price I. R. Zhao Q. (2019). Novel Lysine-Based Thioureas as Mechanism-Based Inhibitors of Sirtuin 2 (SIRT2) with Anticancer Activity in a Colorectal Cancer Murine Model. J. Med. Chem. 62, 4131–4141. 10.1021/acs.jmedchem.9b00191 30986062 PMC6901289

[B44] Feldman J. L. Dittenhafer-Reed K. E. Denu J. M. (2012). Sirtuin Catalysis and Regulation. J. Biol. Chem. 287, 42419–42427. 10.1074/jbc.R112.378877 23086947 PMC3522242

[B45] Feldman J. L. Dittenhafer-Reed K. E. Kudo N. Thelen J. N. Ito A. Yoshida M. (2015). Kinetic and Structural Basis for Acyl-Group Selectivity and NAD(+) Dependence in Sirtuin-Catalyzed Deacylation. Biochemistry 54, 3037–3050. 10.1021/acs.biochem.5b00150 25897714 PMC4470489

[B46] Ferrara G. Benzi A. Sturla L. Marubbi D. Frumento D. Spinelli S. (2020). Sirt6 Inhibition Delays the Onset of Experimental Autoimmune Encephalomyelitis by Reducing Dendritic Cell Migration. J. Neuroinflammation 17, 228. 10.1186/s12974-020-01906-1 32736564 PMC7393881

[B47] Finley L. W. Carracedo A. Lee J. Souza A. Egia A. Zhang J. (2011). SIRT3 Opposes Reprogramming of Cancer Cell Metabolism through HIF1α Destabilization. Cancer Cell 19, 416–428. 10.1016/j.ccr.2011.02.014 21397863 PMC3065720

[B48] Fong Y. Lin Y. C. Wu C. Y. Wang H. M. Lin L. L. Chou H. L. (2014). The Antiproliferative and Apoptotic Effects of Sirtinol, a Sirtuin Inhibitor on Human Lung Cancer Cells by Modulating Akt/β-Catenin-Foxo3a axis. Scientific World Journal 2014, 937051. 10.1155/2014/937051 25184156 PMC4144300

[B49] Fujita Y. Yamashita T. (2018). Sirtuins in Neuroendocrine Regulation and Neurological Diseases. Front. Neurosci. 12, 778. 10.3389/fnins.2018.00778 30416425 PMC6213750

[B50] Funato K. Hayashi T. Echizen K. Negishi L. Shimizu N. Koyama-Nasu R. (2018). SIRT2-mediated Inactivation of P73 Is Required for Glioblastoma Tumorigenicity. EMBO Rep. 19, e45587. 10.15252/embr.201745587 30213795 PMC6216266

[B51] Garcia-Peterson L. M. Ndiaye M. A. Singh C. K. Chhabra G. Huang W. Ahmad N. (2017). SIRT6 Histone Deacetylase Functions as a Potential Oncogene in Human Melanoma. Genes Cancer 8, 701–712. 10.18632/genesandcancer.153 29234488 PMC5724804

[B52] Gertz M. Fischer F. Nguyen G. T. Lakshminarasimhan M. Schutkowski M. Weyand M. (2013). Ex-527 Inhibits Sirtuins by Exploiting Their Unique NAD+-dependent Deacetylation Mechanism. Proc. Natl. Acad. Sci. U. S. A. 110, E2772–E2781. 10.1073/pnas.1303628110 23840057 PMC3725051

[B53] Ghosh A. Sengupta A. Seerapu G. P. K. Nakhi A. Shivaji Ramarao E. V. V. Bung N. (2017). A Novel SIRT1 Inhibitor, 4bb Induces Apoptosis in HCT116 Human colon Carcinoma Cells Partially by Activating P53. Biochem. Biophys. Res. Commun. 488, 562–569. 10.1016/j.bbrc.2017.05.089 28526414

[B54] Giblin W. Bringman-Rodenbarger L. Guo A. H. Kumar S. Monovich A. C. Mostafa A. M. (2021). The Deacylase SIRT5 Supports Melanoma Viability by Influencing Chromatin Dynamics. J. Clin. Invest. 131, e138926. 10.1172/jci138926 PMC8203465 33945506

[B55] Gomes P. Leal H. Mendes A. F. Reis F. Cavadas C. (2019). Dichotomous Sirtuins: Implications for Drug Discovery in Neurodegenerative and Cardiometabolic Diseases. Trends Pharmacol. Sci. 40, 1021–1039. 10.1016/j.tips.2019.09.003 31704173

[B56] Greene K. S. Lukey M. J. Wang X. Blank B. Druso J. E. Lin M. J. (2019). SIRT5 Stabilizes Mitochondrial Glutaminase and Supports Breast Cancer Tumorigenesis. Proc. Natl. Acad. Sci. U. S. A. 116, 26625. 10.1073/pnas.1911954116 PMC6936584 31843902

[B57] Grozinger C. M. Chao E. D. Blackwell H. E. Moazed D. Schreiber S. L. (2001). Identification of a Class of Small Molecule Inhibitors of the Sirtuin Family of NAD-dependent Deacetylases by Phenotypic Screening. J. Biol. Chem. 276, 38837–38843. 10.1074/jbc.M106779200 11483616

[B58] Guo W. Qian L. Zhang J. Zhang W. Morrison A. Hayes P. (2011). Sirt1 Overexpression in Neurons Promotes Neurite Outgrowth and Cell Survival through Inhibition of the mTOR Signaling. J. Neurosci. Res. 89, 1723–1736. 10.1002/jnr.22725 21826702

[B59] Haigis M. C. Sinclair D. A. (2010). Mammalian Sirtuins: Biological Insights and Disease Relevance. Annu. Rev. Pathol. 5, 253–295. 10.1146/annurev.pathol.4.110807.092250 20078221 PMC2866163

[B60] Han M. K. Song E. K. Guo Y. Ou X. Mantel C. Broxmeyer H. E. (2008). SIRT1 Regulates Apoptosis and Nanog Expression in Mouse Embryonic Stem Cells by Controlling P53 Subcellular Localization. Cell Stem Cell 2, 241–251. 10.1016/j.stem.2008.01.002 18371449 PMC2819008

[B61] Heltweg B. Gatbonton T. Schuler A. D. Posakony J. Li H. Goehle S. (2006). Antitumor Activity of a Small-Molecule Inhibitor of Human Silent Information Regulator 2 Enzymes. Cancer Res. 66, 4368–4377. 10.1158/0008-5472.CAN-05-3617 16618762

[B64] Hong J. Y. Fernandez I. Anmangandla A. Lu X. Bai J. J. Lin H. (2021). Pharmacological Advantage of SIRT2-Selective versus Pan-SIRT1-3 Inhibitors. ACS Chem. Biol. 16, 1266–1275. 10.1021/acschembio.1c00331 34139124

[B62] Hong J. Y. Jing H. Price I. R. Cao J. Bai J. J. Lin H. (2020). Simultaneous Inhibition of SIRT2 Deacetylase and Defatty-Acylase Activities via a PROTAC Strategy. ACS Med. Chem. Lett. 11, 2305–2311. 10.1021/acsmedchemlett.0c00423 33214845 PMC7667848

[B65] Howitz K. T. Bitterman K. J. Cohen H. Y. Lamming D. W. Lavu S. Wood J. G. (2003). Small Molecule Activators of Sirtuins Extend Saccharomyces cerevisiae Lifespan. Nature 425, 191–196. 10.1038/nature01960 12939617

[B66] Hu J. Jing H. Lin H. (2014). Sirtuin Inhibitors as Anticancer Agents. Future Med. Chem. 6, 945–966. 10.4155/fmc.14.44 24962284 PMC4384657

[B68] Huang H. Zhang D. Wang Y. Perez-Neut M. Han Z. Zheng Y. G. (2018). Lysine Benzoylation Is a Histone Mark Regulated by SIRT2. Nat. Commun. 9, 3374. 10.1038/s41467-018-05567-w 30154464 PMC6113264

[B67] Huang N. Liu Z. Zhu J. Cui Z. Li Y. Yu Y. (2017). Sirtuin 6 Plays an Oncogenic Role and Induces Cell Autophagy in Esophageal Cancer Cells. Tumour Biol. 39, 1010428317708532. 10.1177/1010428317708532 28653878

[B70] Huang Y. Zhang J. Xu D. Peng Y. Jin Y. Zhang L. (2021). SIRT6 Specific Inhibitor OSS128167 Exacerbates Diabetic Cardiomyopathy by Aggravating Inflammation and Oxidative Stress. Mol. Med. Rep. 23, 367. 10.3892/mmr.2021.12006 33760202 PMC7986000

[B69] Huang Z. Zhao J. Deng W. Chen Y. Shang J. Song K. (2018). Identification of a Cellularly Active SIRT6 Allosteric Activator. Nat. Chem. Biol. 14, 1118–1126. 10.1038/s41589-018-0150-0 30374165

[B72] Hubbard B. P. Gomes A. P. Dai H. Li J. Case A. W. Considine T. (2013). Evidence for a Common Mechanism of SIRT1 Regulation by Allosteric Activators. Science 339, 1216–1219. 10.1126/science.1231097 23471411 PMC3799917

[B71] Hubbard B. P. Sinclair D. A. (2014). Small Molecule SIRT1 Activators for the Treatment of Aging and Age-Related Diseases. Trends Pharmacol. Sci. 35, 146–154. 10.1016/j.tips.2013.12.004 24439680 PMC3970218

[B73] Iachettini S. Trisciuoglio D. Rotili D. Lucidi A. Salvati E. Zizza P. (2018). Pharmacological Activation of SIRT6 Triggers Lethal Autophagy in Human Cancer Cells. Cell Death Dis. 9, 996. 10.1038/s41419-018-1065-0 30250025 PMC6155207

[B74] Igase M. Fujiki N. Shibutani S. Sakai H. Noguchi S. Nemoto Y. (2020). Tenovin-6 Induces the SIRT-independent Cell Growth Suppression and Blocks Autophagy Flux in Canine Hemangiosarcoma Cell Lines. Exp. Cell Res. 388, 111810. 10.1016/j.yexcr.2019.111810 31891684

[B75] Igci M. Kalender M. E. Borazan E. Bozgeyik I. Bayraktar R. Bozgeyik E. (2016). High-throughput Screening of Sirtuin Family of Genes in Breast Cancer. Gene 586, 123–128. 10.1016/j.gene.2016.04.023 27080717

[B76] Iside C. Scafuro M. Nebbioso A. Altucci L. (2020). SIRT1 Activation by Natural Phytochemicals: An Overview. Front. Pharmacol. 11, 1225. 10.3389/fphar.2020.01225 32848804 PMC7426493

[B78] Jeong H. Cohen D. E. Cui L. Supinski A. Savas J. N. Mazzulli J. R. (2011). Sirt1 Mediates Neuroprotection from Mutant Huntingtin by Activation of the TORC1 and CREB Transcriptional Pathway. Nat. Med. 18, 159–165. 10.1038/nm.2559 22179316 PMC3509213

[B77] Jeong S. G. Cho G. W. (2017). The Tubulin Deacetylase Sirtuin-2 Regulates Neuronal Differentiation through the ERK/CREB Signaling Pathway. Biochem. Biophys. Res. Commun. 482, 182–187. 10.1016/j.bbrc.2016.11.031 27838300

[B79] Ji S. Doucette J. R. Nazarali A. J. (2011). Sirt2 Is a Novel *In Vivo* Downstream Target of Nkx2.2 and Enhances Oligodendroglial Cell Differentiation. J. Mol. Cell. Biol. 3, 351–359. 10.1093/jmcb/mjr009 21669943

[B80] Jiang H. Khan S. Wang Y. Charron G. He B. Sebastian C. (2013). SIRT6 Regulates TNF-α Secretion through Hydrolysis of Long-Chain Fatty Acyl Lysine. Nature 496, 110–113. 10.1038/nature12038 23552949 PMC3635073

[B81] Jin J. He B. Zhang X. Lin H. Wang Y. (2016). SIRT2 Reverses 4-Oxononanoyl Lysine Modification on Histones. J. Am. Chem. Soc. 138, 12304–12307. 10.1021/jacs.6b04977 27610633 PMC5305808

[B83] Jing H. Hu J. He B. Negrón Abril Y. L. Stupinski J. Weiser K. (2016). A SIRT2-Selective Inhibitor Promotes C-Myc Oncoprotein Degradation and Exhibits Broad Anticancer Activity. Cancer Cell 29, 297–310. 10.1016/j.ccell.2016.04.005 26977881 PMC4811675

[B82] Jing H. Lin H. (2015). Sirtuins in Epigenetic Regulation. Chem. Rev. 115, 2350–2375. 10.1021/cr500457h 25804908 PMC4610301

[B84] Jing H. Zhang X. Wisner S. A. Chen X. Spiegelman N. A. Linder M. E. (2017). SIRT2 and Lysine Fatty Acylation Regulate the Transforming Activity of K-Ras4a. eLife 6, e32436. 10.7554/eLife.32436 29239724 PMC5745086

[B85] Julien C. Tremblay C. Emond V. Lebbadi M. Salem N. Jr. Bennett D. A. (2009). Sirtuin 1 Reduction Parallels the Accumulation of Tau in Alzheimer Disease. J. Neuropathol. Exp. Neurol. 68, 48–58. 10.1097/NEN.0b013e3181922348 19104446 PMC2813570

[B86] Kaitsuka T. Matsushita M. Matsushita N. (2020). SIRT2 Inhibition Activates Hypoxia-Inducible Factor 1α Signaling and Mediates Neuronal Survival. Biochem. Biophys. Res. Commun. 529, 957–962. 10.1016/j.bbrc.2020.06.159 32819605

[B87] Kaluski S. Portillo M. Besnard A. Stein D. Einav M. Zhong L. (2017). Neuroprotective Functions for the Histone Deacetylase SIRT6. Cell Rep. 18, 3052–3062. 10.1016/j.celrep.2017.03.008 28355558 PMC5389893

[B88] Kawahara T. L. Michishita E. Adler A. S. Damian M. Berber E. Lin M. (2009). SIRT6 Links Histone H3 Lysine 9 Deacetylation to NF-kappaB-dependent Gene Expression and Organismal Life Span. Cell 136, 62–74. 10.1016/j.cell.2008.10.052 19135889 PMC2757125

[B89] Ke X. Qin Q. Deng T. Liao Y. Gao S. J. (2020). Heterogeneous Responses of Gastric Cancer Cell Lines to Tenovin-6 and Synergistic Effect with Chloroquine. Cancers (Basel) 12, 365. 10.3390/cancers12020365 PMC7072542 32033497

[B90] Khan D. Sarikhani M. Dasgupta S. Maniyadath B. Pandit A. S. Mishra S. (2018). SIRT6 Deacetylase Transcriptionally Regulates Glucose Metabolism in Heart. J. Cell. Physiol. 233, 5478–5489. 10.1002/jcp.26434 29319170

[B93] Kim B. S. Lee C. H. Chang G. E. Cheong E. Shin I. (2016). A Potent and Selective Small Molecule Inhibitor of Sirtuin 1 Promotes Differentiation of Pluripotent P19 Cells into Functional Neurons. Sci. Rep. 6, 34324. 10.1038/srep34324 27680533 PMC5041152

[B92] Kim H. B. Lee S. H. Um J. H. Kim M. J. Hyun S. K. Gong E. J. (2015). Sensitization of Chemo-Resistant Human Chronic Myeloid Leukemia Stem-like Cells to Hsp90 Inhibitor by SIRT1 Inhibition. Int. J. Biol. Sci. 11, 923–934. 10.7150/ijbs.10896 26157347 PMC4495410

[B91] Kim H. S. Vassilopoulos A. Wang R. H. Lahusen T. Xiao Z. Xu X. (2011). SIRT2 Maintains Genome Integrity and Suppresses Tumorigenesis through Regulating APC/C Activity. Cancer Cell 20, 487–499. 10.1016/j.ccr.2011.09.004 22014574 PMC3199577

[B94] Kojima K. Ohhashi R. Fujita Y. Hamada N. Akao Y. Nozawa Y. (2008). A Role for SIRT1 in Cell Growth and Chemoresistance in Prostate Cancer PC3 and DU145 Cells. Biochem. Biophys. Res. Commun. 373, 423–428. 10.1016/j.bbrc.2008.06.045 18573234

[B95] Kokkonen P. Rahnasto-Rilla M. Mellini P. Jarho E. Lahtela-Kakkonen M. Kokkola T. (2014). Studying SIRT6 Regulation Using H3K56 Based Substrate and Small Molecules. Eur. J. Pharm. Sci. 63, 71–76. 10.1016/j.ejps.2014.06.015 25004411

[B97] Kosciuk T. Price I. R. Zhang X. Zhu C. Johnson K. N. Zhang S. (2020). NMT1 and NMT2 Are Lysine Myristoyltransferases Regulating the ARF6 GTPase Cycle. Nat. Commun. 11, 1067. 10.1038/s41467-020-14893-x 32103017 PMC7044312

[B96] Kosciuk T. Wang M. Hong J. Y. Lin H. (2019). Updates on the Epigenetic Roles of Sirtuins. Curr. Opin. Chem. Biol. 51, 18–29. 10.1016/j.cbpa.2019.01.023 30875552 PMC6698398

[B98] Kozako T. Aikawa A. Shoji T. Fujimoto T. Yoshimitsu M. Shirasawa S. (2012). High Expression of the Longevity Gene Product SIRT1 and Apoptosis Induction by Sirtinol in Adult T-Cell Leukemia Cells. Int. J. Cancer 131, 2044–2055. 10.1002/ijc.27481 22322739

[B100] Kozako T. Mellini P. Ohsugi T. Aikawa A. Uchida Y. I. Honda S. I. (2018). Novel Small Molecule SIRT2 Inhibitors Induce Cell Death in Leukemic Cell Lines. BMC Cancer 18, 791. 10.1186/s12885-018-4710-1 30081901 PMC6091197

[B99] Kozako T. Suzuki T. Yoshimitsu M. Uchida Y. Kuroki A. Aikawa A. (2015). Novel Small-Molecule SIRT1 Inhibitors Induce Cell Death in Adult T-Cell Leukaemia Cells. Sci. Rep. 5, 11345. 10.1038/srep11345 26091232 PMC4473680

[B101] Kudo N. Ito A. Arata M. Nakata A. Yoshida M. (2018). Identification of a Novel Small Molecule that Inhibits Deacetylase but Not Defatty-Acylase Reaction Catalysed by SIRT2. Philos. Trans. R. Soc. Lond. B Biol. Sci. 373, 20170070. 10.1098/rstb.2017.0070 29685974 PMC5915714

[B102] Kugel S. Sebastián C. Fitamant J. Ross K. N. Saha S. K. Jain E. (2016). SIRT6 Suppresses Pancreatic Cancer through Control of Lin28b. Cell 165, 1401–1415. 10.1016/j.cell.2016.04.033 27180906 PMC4892983

[B103] Kumar S. Lombard D. B. (2017). For Certain, SIRT4 Activities!. Trends Biochem. Sci. 42, 499–501. 10.1016/j.tibs.2017.05.008 28587732 PMC5518788

[B104] Laaroussi H. Ding Y. Teng Y. Deschamps P. Vidal M. Yu P. (2020). Synthesis of Indole Inhibitors of Silent Information Regulator 1 (SIRT1), and Their Evaluation as Cytotoxic Agents. Eur. J. Med. Chem. 202, 112561. 10.1016/j.ejmech.2020.112561 32711231

[B105] Lain S. Hollick J. J. Campbell J. Staples O. D. Higgins M. Aoubala M. (2008). Discovery, *In Vivo* Activity, and Mechanism of Action of a Small-Molecule P53 Activator. Cancer Cell 13, 454–463. 10.1016/j.ccr.2008.03.004 18455128 PMC2742717

[B106] Lara E. Mai A. Calvanese V. Altucci L. Lopez-Nieva P. Martinez-Chantar M. L. (2009). Salermide, a Sirtuin Inhibitor with a strong Cancer-specific Proapoptotic Effect. Oncogene 28, 781–791. 10.1038/onc.2008.436 19060927

[B107] Lasigliè D. Boero S. Bauer I. Morando S. Damonte P. Cea M. (2016). Sirt6 Regulates Dendritic Cell Differentiation, Maturation, and Function. Aging (Albany NY) 8, 34–49. 10.18632/aging.100870 26761436 PMC4761712

[B108] Lee B. B. Kim Y. Kim D. Cho E. Y. Han J. Kim H. K. (2019). Metformin and Tenovin-6 Synergistically Induces Apoptosis through LKB1-independent SIRT1 Down-Regulation in Non-small Cell Lung Cancer Cells. J. Cell Mol. Med. 23, 2872–2889. 10.1111/jcmm.14194 30710424 PMC6433689

[B109] Lee H. C. (2006). Structure and Enzymatic Functions of Human CD38. Mol. Med. 12, 317–323. 10.2119/2006-00086.Lee 17380198 PMC1829193

[B115] Li M. Chiang Y. L. Lyssiotis C. A. Teater M. R. Hong J. Y. Shen H. (2019). Non-oncogene Addiction to SIRT3 Plays a Critical Role in Lymphomagenesis. Cancer Cell 35, 916–e9. 10.1016/j.ccell.2019.05.002 31185214 PMC7534582

[B111] Li S. Banck M. Mujtaba S. Zhou M. M. Sugrue M. M. Walsh M. J. (2010). p53-induced Growth Arrest Is Regulated by the Mitochondrial SirT3 Deacetylase. PLoS One 5, e10486. 10.1371/journal.pone.0010486 20463968 PMC2864751

[B114] Li W. Guo B. Tao K. Li F. Liu Z. Yao H. (2019). Inhibition of SIRT1 in Hippocampal CA1 Ameliorates PTSD-like Behaviors in Mice by Protections of Neuronal Plasticity and Serotonin Homeostasis via NHLH2/MAO-A Pathway. Biochem. Biophys. Res. Commun. 518, 344–350. 10.1016/j.bbrc.2019.08.060 31421827

[B110] Li W. Zhang B. Tang J. Cao Q. Wu Y. Wu C. (2007). Sirtuin 2, a Mammalian Homolog of Yeast Silent Information Regulator-2 Longevity Regulator, Is an Oligodendroglial Protein that Decelerates Cell Differentiation through Deacetylating Alpha-Tubulin. J. Neurosci. 27, 2606–2616. 10.1523/JNEUROSCI.4181-06.2007 17344398 PMC6672490

[B112] Li Y. Meng X. Wang W. Liu F. Hao Z. Yang Y. (2017). Cardioprotective Effects of SIRT6 in a Mouse Model of Transverse Aortic Constriction-Induced Heart Failure. Front. Physiol. 8, 394. 10.3389/fphys.2017.00394 28659816 PMC5468374

[B113] Li Y. Zhang M. Dorfman R. G. Pan Y. Tang D. Xu L. (2018). SIRT2 Promotes the Migration and Invasion of Gastric Cancer through RAS/ERK/JNK/MMP-9 Pathway by Increasing PEPCK1-Related Metabolism. Neoplasia 20, 745–756. 10.1016/j.neo.2018.03.008 29925042 PMC6005814

[B116] Lim J. H. Lee Y. M. Chun Y. S. Chen J. Kim J. E. Park J. W. (2010). Sirtuin 1 Modulates Cellular Responses to Hypoxia by Deacetylating Hypoxia-Inducible Factor 1alpha. Mol. Cell 38, 864–878. 10.1016/j.molcel.2010.05.023 20620956

[B118] Liu L. Arun A. Ellis L. Peritore C. Donmez G. (2014). SIRT2 Enhances 1-Methyl-4-Phenyl-1,2,3,6-Tetrahydropyridine (MPTP)-induced Nigrostriatal Damage via Apoptotic Pathway. Front. Aging Neurosci. 6, 184. 10.3389/fnagi.2014.00184 25157229 PMC4127494

[B117] Liu P. Y. Xu N. Malyukova A. Scarlett C. J. Sun Y. T. Zhang X. D. (2013). The Histone Deacetylase SIRT2 Stabilizes Myc Oncoproteins. Cell Death Differ 20, 503–514. 10.1038/cdd.2012.147 23175188 PMC3569991

[B119] Liu Z. Wang J. Huang X. Li Z. Liu P. (2016). Deletion of Sirtuin 6 Accelerates Endothelial Dysfunction and Atherosclerosis in Apolipoprotein E-Deficient Mice. Transl. Res. 172, 18–e2. 10.1016/j.trsl.2016.02.005 26924042

[B120] Lu B. Zhang D. Wang X. Lin D. Chen Y. Xu X. (2021). Targeting SIRT1 to Inhibit the Proliferation of Multiple Myeloma Cells. Oncol. Lett. 21, 306. 10.3892/ol.2021.12567 33732382 PMC7905587

[B121] Ma W. Zhao X. Wang K. Liu J. Huang G. (2018). Dichloroacetic Acid (DCA) Synergizes with the SIRT2 Inhibitor Sirtinol and AGK2 to Enhance Anti-tumor Efficacy in Non-small Cell Lung Cancer. Cancer Biol. Ther. 19, 835–846. 10.1080/15384047.2018.1480281 30067423 PMC6154844

[B122] Machado de Oliveira R. Sarkander J. Kazantsev A. G. Outeiro T. F. (2012). SIRT2 as a Therapeutic Target for Age-Related Disorders. Front. Pharmacol. 3, 82. 10.3389/fphar.2012.00082 22563317 PMC3342661

[B123] Mai A. Massa S. Lavu S. Pezzi R. Simeoni S. Ragno R. (2005). Design, Synthesis, and Biological Evaluation of Sirtinol Analogues as Class III Histone/protein Deacetylase (Sirtuin) Inhibitors. J. Med. Chem. 48, 7789–7795. 10.1021/jm050100l 16302818

[B124] Mangas-Sanjuan V. Oláh J. Gonzalez-Alvarez I. Lehotzky A. Tőkési N. Bermejo M. (2015). Tubulin Acetylation Promoting Potency and Absorption Efficacy of Deacetylase Inhibitors. Br. J. Pharmacol. 172, 829–840. 10.1111/bph.12946 25257800 PMC4301692

[B125] Marquardt J. U. Fischer K. Baus K. Kashyap A. Ma S. Krupp M. (2013). Sirtuin-6-dependent Genetic and Epigenetic Alterations Are Associated with Poor Clinical Outcome in Hepatocellular Carcinoma Patients. Hepatology 58, 1054–1064. 10.1002/hep.26413 23526469 PMC3759627

[B126] Marshall G. M. Liu P. Y. Gherardi S. Scarlett C. J. Bedalov A. Xu N. (2011). SIRT1 Promotes N-Myc Oncogenesis through a Positive Feedback Loop Involving the Effects of MKP3 and ERK on N-Myc Protein Stability. Plos Genet. 7, e1002135. 10.1371/journal.pgen.1002135 21698133 PMC3116909

[B127] Mathias R. A. Greco T. M. Oberstein A. Budayeva H. G. Chakrabarti R. Rowland E. A. (2014). Sirtuin 4 Is a Lipoamidase Regulating Pyruvate Dehydrogenase Complex Activity. Cell 159, 1615–1625. 10.1016/j.cell.2014.11.046 25525879 PMC4344121

[B128] McCarthy A. R. Pirrie L. Hollick J. J. Ronseaux S. Campbell J. Higgins M. (2012). Synthesis and Biological Characterisation of Sirtuin Inhibitors Based on the Tenovins. Bioorg. Med. Chem. 20, 1779–1793. 10.1016/j.bmc.2012.01.001 22304848

[B130] Mellini P. Itoh Y. Elboray E. E. Tsumoto H. Li Y. Suzuki M. (2019). Identification of Diketopiperazine-Containing 2-Anilinobenzamides as Potent Sirtuin 2 (SIRT2)-Selective Inhibitors Targeting the "Selectivity Pocket", Substrate-Binding Site, and NAD+-Binding Site. J. Med. Chem. 62, 5844–5862. 10.1021/acs.jmedchem.9b00255 31144814

[B129] Mellini P. Itoh Y. Tsumoto H. Li Y. Suzuki M. Tokuda N. (2017). Potent Mechanism-Based Sirtuin-2-Selective Inhibition by an In Situ-generated Occupant of the Substrate-Binding Site, “selectivity Pocket” and NAD+-binding Site. Chem. Sci. 8, 6400–6408. 10.1039/c7sc02738a 28989670 PMC5628579

[B131] Menssen A. Hydbring P. Kapelle K. Vervoorts J. Diebold J. Lüscher B. (2012). The C-MYC Oncoprotein, the NAMPT Enzyme, the SIRT1-Inhibitor DBC1, and the SIRT1 Deacetylase Form a Positive Feedback Loop. Proc. Natl. Acad. Sci. U S A. 109, E187–E196. 10.1073/pnas.1105304109 22190494 PMC3268300

[B132] Michán S. Li Y. Chou M. M. Parrella E. Ge H. Long J. M. (2010). SIRT1 Is Essential for normal Cognitive Function and Synaptic Plasticity. J. Neurosci. 30, 9695–9707. 10.1523/JNEUROSCI.0027-10.2010 20660252 PMC2921958

[B133] Michishita E. McCord R. A. Berber E. Kioi M. Padilla-Nash H. Damian M. (2008). SIRT6 Is a Histone H3 Lysine 9 Deacetylase that Modulates Telomeric Chromatin. Nature 452, 492–496. 10.1038/nature06736 18337721 PMC2646112

[B134] Michishita E. McCord R. A. Boxer L. D. Barber M. F. Hong T. Gozani O. (2009). Cell Cycle-dependent Deacetylation of Telomeric Histone H3 Lysine K56 by Human SIRT6. Cell Cycle 8, 2664–2666. 10.4161/cc.8.16.9367 19625767 PMC4474138

[B135] Milne J. C. Lambert P. D. Schenk S. Carney D. P. Smith J. J. Gagne D. J. (2007). Small Molecule Activators of SIRT1 as Therapeutics for the Treatment of Type 2 Diabetes. Nature 450, 712–716. 10.1038/nature06261 18046409 PMC2753457

[B136] Moniot S. Forgione M. Lucidi A. Hailu G. S. Nebbioso A. Carafa V. (2017). Development of 1,2,4-Oxadiazoles as Potent and Selective Inhibitors of the Human Deacetylase Sirtuin 2: Structure-Activity Relationship, X-ray Crystal Structure, and Anticancer Activity. J. Med. Chem. 60, 2344–2360. 10.1021/acs.jmedchem.6b01609 28240897

[B137] Morris B. J. (2013). Seven Sirtuins for Seven Deadly Diseases of Aging. Free Radic. Biol. Med. 56, 133–171. 10.1016/j.freeradbiomed.2012.10.525 23104101

[B138] Muscolini M. Castiello L. Palermo E. Zevini A. Ferrari M. Olagnier D. (2019). SIRT1 Modulates the Sensitivity of Prostate Cancer Cells to Vesicular Stomatitis Virus Oncolysis. J. Virol. 93. 10.1128/JVI.00626-19 PMC6639275 31092575

[B139] Napper A. D. Hixon J. McDonagh T. Keavey K. Pons J. F. Barker J. (2005). Discovery of Indoles as Potent and Selective Inhibitors of the Deacetylase SIRT1. J. Med. Chem. 48, 8045–8054. 10.1021/jm050522v 16335928

[B140] Nguyen G. T. Schaefer S. Gertz M. Weyand M. Steegborn C. (2013). Structures of Human Sirtuin 3 Complexes with ADP-Ribose and with Carba-Nad+ and SRT1720: Binding Details and Inhibition Mechanism. Acta Crystallogr. D Biol. Crystallogr. 69, 1423–1432. 10.1107/S0907444913015448 23897466

[B141] Nikseresht S. Khodagholi F. Ahmadiani A. (2019). Protective Effects of Ex-527 on Cerebral Ischemia-Reperfusion Injury through Necroptosis Signaling Pathway Attenuation. J. Cell. Physiol. 234, 1816–1826. 10.1002/jcp.27055 30067864

[B142] Nishida Y. Rardin M. J. Carrico C. He W. Sahu A. K. Gut P. (2015). SIRT5 Regulates Both Cytosolic and Mitochondrial Protein Malonylation with Glycolysis as a Major Target. Mol. Cell 59, 321–332. 10.1016/j.molcel.2015.05.022 26073543 PMC4571487

[B143] North B. J. Verdin E. (2007). Interphase Nucleo-Cytoplasmic Shuttling and Localization of SIRT2 during Mitosis. PLoS One 2, e784. 10.1371/journal.pone.0000784 17726514 PMC1949146

[B144] Oon C. E. Strell C. Yeong K. Y. Östman A. Prakash J. (2015). SIRT1 Inhibition in Pancreatic Cancer Models: Contrasting Effects *In Vitro* and *In Vivo* . Eur. J. Pharmacol. 757, 59–67. 10.1016/j.ejphar.2015.03.064 25843411

[B145] Ota H. Tokunaga E. Chang K. Hikasa M. Iijima K. Eto M. (2006). Sirt1 Inhibitor, Sirtinol, Induces Senescence-like Growth Arrest with Attenuated Ras-MAPK Signaling in Human Cancer Cells. Oncogene 25, 176–185. 10.1038/sj.onc.1209049 16170353

[B146] Outeiro T. F. Kontopoulos E. Altmann S. M. Kufareva I. Strathearn K. E. Amore A. M. (2007). Sirtuin 2 Inhibitors rescue Alpha-Synuclein-Mediated Toxicity in Models of Parkinson's Disease. Science 317, 516–519. 10.1126/science.1143780 17588900

[B147] Pacholec M. Bleasdale J. E. Chrunyk B. Cunningham D. Flynn D. Garofalo R. S. (2010). SRT1720, SRT2183, SRT1460, and Resveratrol Are Not Direct Activators of SIRT1. J. Biol. Chem. 285, 8340–8351. 10.1074/jbc.M109.088682 20061378 PMC2832984

[B148] Pannek M. Simic Z. Fuszard M. Meleshin M. Rotili D. Mai A. (2017). Crystal Structures of the Mitochondrial Deacylase Sirtuin 4 Reveal Isoform-specific Acyl Recognition and Regulation Features. Nat. Commun. 8, 1513. 10.1038/s41467-017-01701-2 29138502 PMC5686155

[B149] Parenti M. D. Grozio A. Bauer I. Galeno L. Damonte P. Millo E. (2014). Discovery of Novel and Selective SIRT6 Inhibitors. J. Med. Chem. 57, 4796–4804. 10.1021/jm500487d 24785705

[B150] Plaitakis A. Kalef-Ezra E. Kotzamani D. Zaganas I. Spanaki C. (2017). The Glutamate Dehydrogenase Pathway and its Roles in Cell and Tissue Biology in Health and Disease. Biology (Basel) 6, 11. 10.3390/biology6010011 PMC5372004 28208702

[B151] Portmann S. Fahrner R. Lechleiter A. Keogh A. Overney S. Laemmle A. (2013). Antitumor Effect of SIRT1 Inhibition in Human HCC Tumor Models *In Vitro* and *In Vivo* . Mol. Cancer Ther. 12, 499–508. 10.1158/1535-7163.MCT-12-0700 23339189

[B152] Prozorovski T. Schulze-Topphoff U. Glumm R. Baumgart J. Schröter F. Ninnemann O. (2008). Sirt1 Contributes Critically to the Redox-dependent Fate of Neural Progenitors. Nat. Cell Biol. 10, 385–394. 10.1038/ncb1700 18344989

[B154] Ren M. Yang X. Bie J. Wang Z. Liu M. Li Y. (2020). Citrate Synthase Desuccinylation by SIRT5 Promotes colon Cancer Cell Proliferation and Migration. Biol. Chem. 401, 1031. 10.1515/hsz-2020-0118 32284438

[B153] Ren N. S. X. Ji M. Tokar E. J. Busch E. L. Xu X. Lewis D. (2017). Haploinsufficiency of SIRT1 Enhances Glutamine Metabolism and Promotes Cancer Development. Curr. Biol. 27, 483–494. 10.1016/j.cub.2016.12.047 28162896 PMC5319916

[B155] Rumpf T. Schiedel M. Karaman B. Roessler C. North B. J. Lehotzky A. (2015). Selective Sirt2 Inhibition by Ligand-Induced Rearrangement of the Active Site. Nat. Commun. 6, 6263. 10.1038/ncomms7263 25672491 PMC4339887

[B156] Safari F. Shekarforoosh S. Hashemi T. Namvar Aghdash S. Fekri A. Safari F. (2017). Sirtinol Abrogates Late Phase of Cardiac Ischemia Preconditioning in Rats. J. Physiol. Sci. 67, 515–522. 10.1007/s12576-016-0483-y 27677982 PMC10717902

[B157] Salehi B. Mishra A. P. Nigam M. Sener B. Kilic M. Sharifi-Rad M. (2018). Resveratrol: A Double-Edged Sword in Health Benefits. Biomedicines 6, 91. 10.3390/biomedicines6030091 PMC6164842 30205595

[B158] Sauve A. A. Celic I. Avalos J. Deng H. Boeke J. D. Schramm V. L. (2001). Chemistry of Gene Silencing: the Mechanism of NAD+-dependent Deacetylation Reactions. Biochemistry 40, 15456–15463. 11747420 10.1021/bi011858j

[B159] Sauve A. A. Wolberger C. Schramm V. L. Boeke J. D. (2006). The Biochemistry of Sirtuins. Annu. Rev. Biochem. 75, 435–465. 10.1146/annurev.biochem.74.082803.133500 16756498

[B160] Schnekenburger M. Goffin E. Lee J. Y. Jang J. Y. Mazumder A. Ji S. (2017). Discovery and Characterization of R/S-N-3-Cyanophenyl-N'-(6-tert-butoxycarbonylamino-3,4-dihydro-2,2-dimethyl-2H-1-benzopyran-4-yl)urea, a New Histone Deacetylase Class III Inhibitor Exerting Antiproliferative Activity against Cancer Cell Lines. J. Med. Chem. 60, 4714–4733. 10.1021/acs.jmedchem.7b00533 28475330

[B161] Sebastián C. Zwaans B. M. Silberman D. M. Gymrek M. Goren A. Zhong L. (2012). The Histone Deacetylase SIRT6 Is a Tumor Suppressor that Controls Cancer Metabolism. Cell 151, 1185–1199. 10.1016/j.cell.2012.10.047 23217706 PMC3526953

[B162] Seifert T. Malo M. Kokkola T. Engen K. Fridén-Saxin M. Wallén E. A. (2014). Chroman-4-one- and Chromone-Based Sirtuin 2 Inhibitors with Antiproliferative Properties in Cancer Cells. J. Med. Chem. 57, 9870–9888. 10.1021/jm500930h 25383691

[B163] Serrano L. Martínez-Redondo P. Marazuela-Duque A. Vazquez B. N. Dooley S. J. Voigt P. (2013). The Tumor Suppressor SirT2 Regulates Cell Cycle Progression and Genome Stability by Modulating the Mitotic Deposition of H4K20 Methylation. Genes Dev. 27, 639–653. 10.1101/gad.211342.112 23468428 PMC3613611

[B164] Shah A. A. Ito A. Nakata A. Yoshida M. (2016). Identification of a Selective SIRT2 Inhibitor and its Anti-breast Cancer Activity. Biol. Pharm. Bull 39, 1739–1742. 10.1248/bpb.b16-00520 27725455

[B165] Shang J. L. Ning S. B. Chen Y. Y. Chen T. X. Zhang J. (2021). MDL-800, an Allosteric Activator of SIRT6, Suppresses Proliferation and Enhances EGFR-TKIs Therapy in Non-small Cell Lung Cancer. Acta Pharmacol. Sin. 42, 120–131. 10.1038/s41401-020-0442-2 32541922 PMC7921659

[B166] She D. T. Wong L. J. Baik S. H. Arumugam T. V. (2018). SIRT2 Inhibition Confers Neuroprotection by Downregulation of FOXO3a and MAPK Signaling Pathways in Ischemic Stroke. Mol. Neurobiol. 55, 9188–9203. 10.1007/s12035-018-1058-0 29654491

[B167] Shi L. Yan H. An S. Shen M. Jia W. Zhang R. (2019). SIRT5-mediated Deacetylation of LDHB Promotes Autophagy and Tumorigenesis in Colorectal Cancer. Mol. Oncol. 13, 358–375. 10.1002/1878-0261.12408 30443978 PMC6360364

[B168] Simic P. Williams E. O. Bell E. L. Gong J. J. Bonkowski M. Guarente L. (2013). SIRT1 Suppresses the Epithelial-To-Mesenchymal Transition in Cancer Metastasis and Organ Fibrosis. Cell Rep. 3, 1175–1186. 10.1016/j.celrep.2013.03.019 23583181 PMC3881428

[B169] Smith B. C. Denu J. M. (2007). Mechanism-Based Inhibition of Sir2 Deacetylases by Thioacetyl-Lysine Peptide. Biochemistry 46, 14478–14486. 10.1021/bi7013294 18027980

[B170] Sociali G. Galeno L. Parenti M. D. Grozio A. Bauer I. Passalacqua M. (2015). Quinazolinedione SIRT6 Inhibitors Sensitize Cancer Cells to Chemotherapeutics. Eur. J. Med. Chem. 102, 530–539. 10.1016/j.ejmech.2015.08.024 26310895

[B171] Sociali G. Magnone M. Ravera S. Damonte P. Vigliarolo T. Von Holtey M. (2017). Pharmacological Sirt6 Inhibition Improves Glucose Tolerance in a Type 2 Diabetes Mouse Model. FASEB J. 31, 3138–3149. 10.1096/fj.201601294R 28386046 PMC6137498

[B172] Solomon J. M. Pasupuleti R. Xu L. McDonagh T. Curtis R. DiStefano P. S. (2006). Inhibition of SIRT1 Catalytic Activity Increases P53 Acetylation but Does Not Alter Cell Survival Following DNA Damage. Mol. Cell. Biol. 26, 28–38. 10.1128/MCB.26.1.28-38.2006 16354677 PMC1317617

[B174] Spiegelman N. A. Hong J. Y. Hu J. Jing H. Wang M. Price I. R. (2019). A Small-Molecule SIRT2 Inhibitor that Promotes K-Ras4a Lysine Fatty-Acylation. ChemMedChem 14, 744–748. 10.1002/cmdc.201800715 30734528 PMC6452895

[B173] Spiegelman N. A. Price I. R. Jing H. Wang M. Yang M. Cao J. (2018). Direct Comparison of SIRT2 Inhibitors: Potency, Specificity, Activity-dependent Inhibition, and On-Target Anticancer Activities. ChemMedChem. 13, 1890–1894. 10.1002/cmdc.201800391 30058233 PMC6402572

[B175] Sundaresan N. R. Vasudevan P. Zhong L. Kim G. Samant S. Parekh V. (2012). The Sirtuin SIRT6 Blocks IGF-Akt Signaling and Development of Cardiac Hypertrophy by Targeting C-Jun. Nat. Med. 18, 1643–1650. 10.1038/nm.2961 23086477 PMC4401084

[B176] Süssmuth S. D. Haider S. Landwehrmeyer G. B. Farmer R. Frost C. Tripepi G. (2015). An Exploratory Double-Blind, Randomized Clinical Trial with Selisistat, a SirT1 Inhibitor, in Patients with Huntington's Disease. Br. J. Clin. Pharmacol. 79, 465–476. 10.1111/bcp.12512 25223731 PMC4345957

[B177] Suzuki T. Khan M. N. Sawada H. Imai E. Itoh Y. Yamatsuta K. (2012). Design, Synthesis, and Biological Activity of a Novel Series of Human Sirtuin-2-Selective Inhibitors. J. Med. Chem. 55, 5760–5773. 10.1021/jm3002108 22642300

[B179] Tan Y. J. Lee Y. T. Petersen S. H. Kaur G. Kono K. Tan S. C. (2019). BZD9L1 Sirtuin Inhibitor as a Potential Adjuvant for Sensitization of Colorectal Cancer Cells to 5-fluorouracil. Ther. Adv. Med. Oncol. 11, 1758835919878977. 10.1177/1758835919878977 31632470 PMC6767736

[B178] Tan Y. J. Lee Y. T. Yeong K. Y. Petersen S. H. Kono K. Tan S. C. (2018). Anticancer Activities of a Benzimidazole Compound through Sirtuin Inhibition in Colorectal Cancer. Future Med. Chem. 10, 2039–2057. 10.4155/fmc-2018-0052 30066578

[B180] Tanno M. Sakamoto J. Miura T. Shimamoto K. Horio Y. (2007). Nucleocytoplasmic Shuttling of the NAD+-dependent Histone Deacetylase SIRT1. J. Biol. Chem. 282, 6823–6832. 10.1074/jbc.M609554200 17197703

[B181] Tasselli L. Xi Y. Zheng W. Tennen R. I. Odrowaz Z. Simeoni F. (2016). SIRT6 Deacetylates H3K18ac at Pericentric Chromatin to Prevent Mitotic Errors and Cellular Senescence. Nat. Struct. Mol. Biol. 23, 434–440. 10.1038/nsmb.3202 27043296 PMC5826646

[B182] Taylor D. M. Balabadra U. Xiang Z. Woodman B. Meade S. Amore A. (2011). A Brain-Permeable Small Molecule Reduces Neuronal Cholesterol by Inhibiting Activity of Sirtuin 2 Deacetylase. ACS Chem. Biol. 6, 540–546. 10.1021/cb100376q 21370928

[B183] Teng Y. B. Jing H. Aramsangtienchai P. He B. Khan S. Hu J. (2015). Efficient Demyristoylase Activity of SIRT2 Revealed by Kinetic and Structural Studies. Sci. Rep. 5, 8529. 10.1038/srep08529 25704306 PMC4894398

[B184] Tong Z. Wang M. Wang Y. Kim D. D. Grenier J. K. Cao J. (2017). SIRT7 Is an RNA-Activated Protein Lysine Deacylase. ACS Chem. Biol. 12, 300–310. 10.1021/acschembio.6b00954 27997115 PMC5326686

[B185] Torrens-Mas M. Oliver J. Roca P. Sastre-Serra J. (2017). SIRT3: Oncogene and Tumor Suppressor in Cancer. Cancers (Basel) 9, 90. 10.3390/cancers9070090 PMC5532626 28704962

[B186] Trammell S. A. Schmidt M. S. Weidemann B. J. Redpath P. Jaksch F. Dellinger R. W. (2016). Nicotinamide Riboside Is Uniquely and Orally Bioavailable in Mice and Humans. Nat. Commun. 7, 12948. 10.1038/ncomms12948 27721479 PMC5062546

[B187] Vikram A. Lewarchik C. M. Yoon J. Y. Naqvi A. Kumar S. Morgan G. M. (2017). Sirtuin 1 Regulates Cardiac Electrical Activity by Deacetylating the Cardiac Sodium Channel. Nat. Med. 23, 361–367. 10.1038/nm.4284 28191886 PMC6218171

[B189] Wang B. Zhang Y. Cao W. Wei X. Chen J. Ying W. (2016). SIRT2 Plays Significant Roles in Lipopolysaccharides-Induced Neuroinflammation and Brain Injury in Mice. Neurochem. Res. 41, 2490–2500. 10.1007/s11064-016-1981-2 27350577

[B188] Wang M. Lin H. (2021). Understanding the Function of Mammalian Sirtuins and Protein Lysine Acylation. Annu. Rev. Biochem. 90, 245. 10.1146/annurev-biochem-082520-125411 33848425

[B190] Wang T. Li X. Sun S. L. (2020). EX527, a Sirt-1 Inhibitor, Induces Apoptosis in Glioma via Activating the P53 Signaling Pathway. Anticancer Drugs 31, 19–26. 10.1097/CAD.0000000000000824 31490284

[B191] Wei L. Liu B. Yao Z. Yuan T. Wang C. Zhang R. (2021). Sirtuin 1 Inhibitor EX527 Suppresses Morphine-Induced Behavioral Sensitization. Neurosci. Lett. 744, 135599. 10.1016/j.neulet.2020.135599 33412237

[B192] Wu D. Lu W. Wei Z. Xu M. Liu X. (2018). Neuroprotective Effect of Sirt2-specific Inhibitor AK-7 against Acute Cerebral Ischemia Is P38 Activation-dependent in Mice. Neuroscience 374, 61–69. 10.1016/j.neuroscience.2018.01.040 29382550

[B193] Wu Z. Zhang Y. Zhang Y. Zhao P. (2020). Sirtuin 2 Inhibition Attenuates Sevoflurane-Induced Learning and Memory Deficits in Developing Rats via Modulating Microglial Activation. Cell Mol. Neurobiol. 40, 437–446. 10.1007/s10571-019-00746-9 31713761 PMC11449016

[B194] Xiangyun Y. Xiaomin N. Linping G. Yunhua X. Ziming L. Yongfeng Y. (2017). Desuccinylation of Pyruvate Kinase M2 by SIRT5 Contributes to Antioxidant Response and Tumor Growth. Oncotarget 8, 6984–6993. 10.18632/oncotarget.14346 28036303 PMC5351684

[B195] Xu Y. Qin Q. Chen R. Wei C. Mo Q. (2018). SIRT1 Promotes Proliferation, Migration, and Invasion of Breast Cancer Cell Line MCF-7 by Upregulating DNA Polymerase delta1 (POLD1). Biochem. Biophys. Res. Commun. 502, 351–357. 10.1016/j.bbrc.2018.05.164 29807012

[B199] Yang H. Chen Y. Jiang Y. Wang D. Yan J. Zhou Z. (2020). TP53 Mutation Influences the Efficacy of Treatment of Colorectal Cancer Cell Lines with a Combination of Sirtuin Inhibitors and Chemotherapeutic Agents. Exp. Ther. Med. 20, 1415–1422. 10.3892/etm.2020.8818 32742376 PMC7388297

[B198] Yang L. L. Wang H. L. Zhong L. Yuan C. Liu S. Y. Yu Z. J. (2018). X-ray crystal Structure Guided Discovery of New Selective, Substrate-Mimicking Sirtuin 2 Inhibitors that Exhibit Activities against Non-small Cell Lung Cancer Cells. Eur. J. Med. Chem. 155, 806–823. 10.1016/j.ejmech.2018.06.041 29957526

[B197] Yang M. H. Laurent G. Bause A. S. Spang R. German N. Haigis M. C. (2013). HDAC6 and SIRT2 Regulate the Acetylation State and Oncogenic Activity of Mutant K-RAS. Mol. Cancer Res. 11, 1072–1077. 10.1158/1541-7786.MCR-13-0040-T 23723075 PMC3778089

[B196] Yang Y. Hou H. Haller E. M. Nicosia S. V. Bai W. (2005). Suppression of FOXO1 Activity by FHL2 through SIRT1-Mediated Deacetylation. EMBO J. 24, 1021–1032. 10.1038/sj.emboj.7600570 15692560 PMC554122

[B200] Yeung F. Hoberg J. E. Ramsey C. S. Keller M. D. Jones D. R. Frye R. A. (2004). Modulation of NF-kappaB-dependent Transcription and Cell Survival by the SIRT1 Deacetylase. EMBO J. 23, 2369–2380. 10.1038/sj.emboj.7600244 15152190 PMC423286

[B201] Yoon Y. K. Ali M. A. Wei A. C. Choon T. S. Shirazi A. N. Parang K. (2015). Discovery of a Potent and Highly Fluorescent Sirtuin Inhibitor. Med. Chem. Commun. 6, 1857–1863. 10.1039/C5MD00307E

[B202] You W. Rotili D. Li T. M. Kambach C. Meleshin M. Schutkowski M. (2017). Structural Basis of Sirtuin 6 Activation by Synthetic Small Molecules. Angew. Chem. Int. Ed. Engl. 56, 1007–1011. 10.1002/anie.201610082 27990725

[B203] Yousafzai N. A. Zhou Q. Xu W. Shi Q. Xu J. Feng L. (2019). SIRT1 Deacetylated and Stabilized XRCC1 to Promote Chemoresistance in Lung Cancer. Cell Death Dis. 10, 363. 10.1038/s41419-019-1592-3 31043584 PMC6494911

[B204] Yuan H. He M. Cheng F. Bai R. da Silva S. R. Aguiar R. C. (2017). Tenovin-6 Inhibits Proliferation and Survival of Diffuse Large B-Cell Lymphoma Cells by Blocking Autophagy. Oncotarget 8, 14912–14924. 10.18632/oncotarget.14741 28118604 PMC5362454

[B206] Zhang G. Liu Z. Qin S. Li K. (2015). Decreased Expression of SIRT6 Promotes Tumor Cell Growth Correlates Closely with Poor Prognosis of Ovarian Cancer. Eur. J. Gynaecol. Oncol. 36, 629–632. 26775341

[B205] Zhang Q. J. Wang Z. Chen H. Z. Zhou S. Zheng W. Liu G. (2008). Endothelium-specific Overexpression of Class III Deacetylase SIRT1 Decreases Atherosclerosis in Apolipoprotein E-Deficient Mice. Cardiovasc. Res. 80, 191–199. 10.1093/cvr/cvn224 18689793 PMC3657473

[B209] Zhang R. Wang C. Tian Y. Yao Y. Mao J. Wang H. (2019). SIRT5 Promotes Hepatocellular Carcinoma Progression by Regulating Mitochondrial Apoptosis. J. Cancer 10, 3871–3882. 10.7150/jca.31266 31333804 PMC6636294

[B207] Zhang X. Khan S. Jiang H. Antonyak M. A. Chen X. Spiegelman N. A. (2016). Identifying the Functional Contribution of the Defatty-Acylase Activity of SIRT6. Nat. Chem. Biol. 12, 614–620. 10.1038/nchembio.2106 27322069 PMC4955683

[B208] Zhang X. Spiegelman N. A. Nelson O. D. Jing H. Lin H. (2017). SIRT6 Regulates Ras-Related Protein R-Ras2 by Lysine Defatty-Acylation. eLife 6, e25158. 10.7554/eLife.25158 28406396 PMC5391209

[B211] Zhao D. Zou S. W. Liu Y. Zhou X. Mo Y. Wang P. (2013). Lysine-5 Acetylation Negatively Regulates Lactate Dehydrogenase A and Is Decreased in Pancreatic Cancer. Cancer Cell 23, 464–476. 10.1016/j.ccr.2013.02.005 23523103 PMC3885615

[B210] Zhao Y. Yang J. Liao W. Liu X. Zhang H. Wang S. (2010). Cytosolic FoxO1 Is Essential for the Induction of Autophagy and Tumour Suppressor Activity. Nat. Cell Biol. 12, 665–675. 10.1038/ncb2069 20543840

[B213] Zhou W. Ni T. K. Wronski A. Glass B. Skibinski A. Beck A. (2016). The SIRT2 Deacetylase Stabilizes Slug to Control Malignancy of Basal-like Breast Cancer. Cell Rep. 17, 1302–1317. 10.1016/j.celrep.2016.10.006 27783945 PMC5108094

[B212] Zhou X. M. Zhang X. Zhang X. S. Zhuang Z. Li W. Sun Q. (2014). SIRT1 Inhibition by Sirtinol Aggravates Brain Edema after Experimental Subarachnoid Hemorrhage. J. Neurosci. Res. 92, 714–722. 10.1002/jnr.23359 24482345

